# Supplementary material for: The quantitative genetics of gene expression in Mimulus guttatus
Source: PLoS Genet. 2024 Apr 11;20(4):e1011072. doi: 10.1371/journal.pgen.1011072 (PMC11060551; doi:10.1371/journal.pgen.1011072)
Supplement: S1 Table — N50 = the length of the shortest scaffold in the ranked list that covers at least 50% of the assembly. (DOCX) [file pgen.1011072.s001.docx]

| Genome | Contig number | Total length of contigs | N50 | BUSCO gene completeness |
| --- | --- | --- | --- | --- |
| 155 | 670 | 295 Mb | 8,107,632 | 93.50% |
| 444 | 1,010 | 323 Mb | 9,112,407 | 93.70% |
| 502 | 134 | 263 Mb | 8,590,551 | 93.70% |
| 541 | 668 | 327 Mb | 9,013,783 | 93.70% |
| 664 | 772 | 300 Mb | 8,222,438 | 93.30% |
| 909 | 1,096 | 311 Mb | 9,340,804 | 92.90% |
| 1034 | 2,953 | 325 Mb | 7,166,111 | 93.20% |
| 1192 | 484 | 284 Mb | 8,422,280 | 93.30% |

**Supplemental Table 1. A summary of features from our *de novo* assemblies based on PacBio sequencing. N50 = the length of the shortest scaffold in the ranked list that covers at least 50 % of the assembly.**
