# Supplementary material for: The quantitative genetics of gene expression in Mimulus guttatus
Source: PLoS Genet. 2024 Apr 11;20(4):e1011072. doi: 10.1371/journal.pgen.1011072 (PMC11060551; doi:10.1371/journal.pgen.1011072)
Supplement: S5 Table — Each predictor was standardized to unit variance (z transform) to make the regression coefficients comparable. (DOCX) [file pgen.1011072.s005.docx]

**Supplemental Table 5. A regression of LRT1 values onto nucleotide diversity (p) and indel diversity (U) within three regions about each gene. Each predictor was standardized to unit variance (z transform) to make the regression coefficients comparable.** **zpi is standardized pi, zI1 is standardized U and (up, genic, down) refers to location. The Regression Equation:**

| LRT1 | = | 70.925 + 8.010 zpi_genic + 1.782 zpi_up + 1.218 zpi_down + 3.801 zI1_genic+ 5.732 zI1_up + 2.185 zI1_down |
| --- | --- | --- |

**Coefficients**

| **Term** | **Coef** | **SE Coef** | **T-Value** | **P-Value** | **VIF** |
| --- | --- | --- | --- | --- | --- |
| Constant | 70.925 | 0.601 | 118.04 | 0.000 |  |
| zpi_genic | 8.010 | 0.813 | 9.85 | 0.000 | 1.83 |
| zpi_up | 1.782 | 0.663 | 2.69 | 0.007 | 1.22 |
| zpi_down | 1.218 | 0.723 | 1.69 | 0.092 | 1.45 |
| zI1_genic | 3.801 | 0.694 | 5.48 | 0.000 | 1.33 |
| zI1_up | 5.732 | 0.635 | 9.03 | 0.000 | 1.10 |
| zI1_down | 2.185 | 0.645 | 3.39 | 0.001 | 1.14 |

**Analysis of Variance**

| **Source** | **DF** | **Adj SS** | **Adj MS** | **F-Value** | **P-Value** |
| --- | --- | --- | --- | --- | --- |
| Regression | 6 | 3042529 | 507088 | 108.26 | 0.000 |
| zpi_genic | 1 | 454633 | 454633 | 97.06 | 0.000 |
| zpi_up | 1 | 33846 | 33846 | 7.23 | 0.007 |
| zpi_down | 1 | 13309 | 13309 | 2.84 | 0.092 |
| zI1_genic | 1 | 140468 | 140468 | 29.99 | 0.000 |
| zI1_up | 1 | 381603 | 381603 | 81.47 | 0.000 |
| zI1_down | 1 | 53780 | 53780 | 11.48 | 0.001 |
| Error | 12968 | 60744257 | 4684 |  |  |
| Total | 12974 | 63786787 |  |  |  |
