# Supplementary material for: The quantitative genetics of gene expression in Mimulus guttatus
Source: PLoS Genet. 2024 Apr 11;20(4):e1011072. doi: 10.1371/journal.pgen.1011072 (PMC11060551; doi:10.1371/journal.pgen.1011072)
Supplement: S7 Table — (DOCX) [file pgen.1011072.s007.docx]

**Supplemental Table 5. The estimated environment (Ve) and genetic (Vg) variance for each PC score is reported for the first 200 PCs. h^2^  the ratio Vg/(Vg+Ve).**

| PC | Ve | Vg | h^2^ |
| --- | --- | --- | --- |
|  |  |  |  |
| 1 | 864.68 | 21.6256 | 0.02 |
| 2 | 651.229 | 76.0974 | 0.10 |
| 3 | 489.081 | 86.8464 | 0.15 |
| 4 | 432.537 | 39.5211 | 0.08 |
| 5 | 271.395 | 28.1904 | 0.09 |
| 6 | 159.537 | 36.5191 | 0.19 |
| 7 | 93.2191 | 32.2605 | 0.26 |
| 8 | 77.854 | 24.5216 | 0.24 |
| 9 | 65.4195 | 25.0404 | 0.28 |
| 10 | 55.8176 | 23.5922 | 0.30 |
| 11 | 26.4773 | 20.393 | 0.44 |
| 12 | 19.8958 | 16.0963 | 0.45 |
| 13 | 39.4762 | 16.9045 | 0.30 |
| 14 | 25.3615 | 16.8053 | 0.40 |
| 15 | 42.4339 | 16.3671 | 0.28 |
| 16 | 48.3534 | 15.8682 | 0.25 |
| 17 | 55.8241 | 32.1848 | 0.37 |
| 18 | 45.5043 | 17.9053 | 0.28 |
| 19 | 70.793 | 10.0213 | 0.12 |
| 20 | 41.2034 | 16.9701 | 0.29 |
| 21 | 49.0364 | 16.0394 | 0.25 |
| 22 | 48.5814 | 9.45625 | 0.16 |
| 23 | 31.5355 | 12.6172 | 0.29 |
| 24 | 45.6123 | 7.37258 | 0.14 |
| 25 | 39.7389 | 7.82757 | 0.16 |
| 26 | 39.9434 | 3.12471 | 0.07 |
| 27 | 35.5009 | 6.94929 | 0.16 |
| 28 | 35.5204 | 4.54212 | 0.11 |
| 29 | 30.9704 | 7.4443 | 0.19 |
| 30 | 28.9775 | 8.93432 | 0.24 |
| 31 | 27.7239 | 8.21527 | 0.23 |
| 32 | 29.0777 | 4.78836 | 0.14 |
| 33 | 21.1136 | 21.5313 | 0.50 |
| 34 | 24.8584 | 5.20177 | 0.17 |
| 35 | 21.8 | 10.5795 | 0.33 |
| 36 | 23.5849 | 6.33759 | 0.21 |
| 37 | 21.7908 | 6.8315 | 0.24 |
| 38 | 21.9556 | 6.4278 | 0.23 |
| 39 | 19.8463 | 6.42892 | 0.24 |
| 40 | 14.0679 | 10.2452 | 0.42 |
| 41 | 19.632 | 5.22447 | 0.21 |
| 42 | 18.845 | 4.88342 | 0.21 |
| 43 | 19.3823 | 3.76363 | 0.16 |
| 44 | 13.2716 | 9.50194 | 0.42 |
| 45 | 14.3651 | 9.60764 | 0.40 |
| 46 | 13.9044 | 8.30503 | 0.37 |
| 47 | 14.1591 | 6.0965 | 0.30 |
| 48 | 18.1143 | 2.73343 | 0.13 |
| 49 | 14.6019 | 5.74715 | 0.28 |
| 50 | 12.4201 | 8.4379 | 0.40 |
| 51 | 11.1849 | 8.90429 | 0.44 |
| 52 | 12.2864 | 8.25115 | 0.40 |
| 53 | 14.0592 | 6.80516 | 0.33 |
| 54 | 14.7869 | 4.51448 | 0.23 |
| 55 | 13.0376 | 5.07057 | 0.28 |
| 56 | 11.6348 | 8.90296 | 0.43 |
| 57 | 13.1348 | 5.60014 | 0.30 |
| 58 | 10.1548 | 8.11115 | 0.44 |
| 59 | 12.541 | 5.69026 | 0.31 |
| 60 | 10.5306 | 8.89698 | 0.46 |
| 61 | 12.6069 | 5.30615 | 0.30 |
| 62 | 13.8603 | 3.40164 | 0.20 |
| 63 | 11.959 | 6.51562 | 0.35 |
| 64 | 10.6148 | 6.46336 | 0.38 |
| 65 | 9.33116 | 8.40322 | 0.47 |
| 66 | 11.5607 | 6.13812 | 0.35 |
| 67 | 8.97361 | 8.46071 | 0.49 |
| 68 | 11.0954 | 5.99772 | 0.35 |
| 69 | 11.3355 | 6.39532 | 0.36 |
| 70 | 10.7406 | 5.84897 | 0.35 |
| 71 | 11.1221 | 5.65429 | 0.34 |
| 72 | 11.2034 | 5.68902 | 0.34 |
| 73 | 11.6034 | 4.8123 | 0.29 |
| 74 | 10.449 | 6.45472 | 0.38 |
| 75 | 11.4748 | 5.0403 | 0.31 |
| 76 | 10.636 | 6.52147 | 0.38 |
| 77 | 10.301 | 6.47893 | 0.39 |
| 78 | 11.7417 | 4.38329 | 0.27 |
| 79 | 9.4066 | 6.73412 | 0.42 |
| 80 | 10.5896 | 6.43387 | 0.38 |
| 81 | 11.276 | 4.40638 | 0.28 |
| 82 | 11.4364 | 3.90428 | 0.25 |
| 83 | 10.3465 | 4.94669 | 0.32 |
| 84 | 8.87506 | 6.9641 | 0.44 |
| 85 | 11.7464 | 2.8081 | 0.19 |
| 86 | 10.1738 | 5.35085 | 0.34 |
| 87 | 10.7818 | 5.0942 | 0.32 |
| 88 | 10.7463 | 4.39625 | 0.29 |
| 89 | 10.6028 | 4.64955 | 0.30 |
| 90 | 11.4965 | 2.92799 | 0.20 |
| 91 | 10.0815 | 5.03053 | 0.33 |
| 92 | 9.44544 | 6.74315 | 0.42 |
| 93 | 10.7188 | 4.30206 | 0.29 |
| 94 | 10.072 | 5.38309 | 0.35 |
| 95 | 10.8772 | 3.64832 | 0.25 |
| 96 | 9.64056 | 5.78066 | 0.37 |
| 97 | 9.61978 | 5.48101 | 0.36 |
| 98 | 9.58873 | 5.14434 | 0.35 |
| 99 | 10.9209 | 2.79911 | 0.20 |
| 100 | 10.1126 | 4.47533 | 0.31 |
| 101 | 10.6454 | 3.67556 | 0.26 |
| 102 | 10.3688 | 3.96935 | 0.28 |
| 103 | 11.2529 | 1.87566 | 0.14 |
| 104 | 10.265 | 4.02586 | 0.28 |
| 105 | 10.2394 | 3.50898 | 0.26 |
| 106 | 10.5269 | 3.16908 | 0.23 |
| 107 | 10.3972 | 3.21795 | 0.24 |
| 108 | 9.95018 | 4.13776 | 0.29 |
| 109 | 8.91225 | 6.54824 | 0.42 |
| 110 | 9.06323 | 5.7428 | 0.39 |
| 111 | 10.5112 | 2.54638 | 0.20 |
| 112 | 9.98805 | 3.50077 | 0.26 |
| 113 | 9.83861 | 4.42517 | 0.31 |
| 114 | 9.50768 | 4.59063 | 0.33 |
| 115 | 9.50453 | 4.70124 | 0.33 |
| 116 | 10.5049 | 2.19631 | 0.17 |
| 117 | 9.90177 | 3.42157 | 0.26 |
| 118 | 10.1994 | 2.51491 | 0.20 |
| 119 | 10.0705 | 3.06271 | 0.23 |
| 120 | 10.1209 | 2.82024 | 0.22 |
| 121 | 9.95232 | 3.05422 | 0.23 |
| 122 | 9.83929 | 3.11158 | 0.24 |
| 123 | 9.99385 | 2.82451 | 0.22 |
| 124 | 10.0301 | 2.90687 | 0.22 |
| 125 | 9.38564 | 3.93266 | 0.30 |
| 126 | 8.96715 | 4.38023 | 0.33 |
| 127 | 10.0264 | 2.62199 | 0.21 |
| 128 | 9.96371 | 2.37977 | 0.19 |
| 129 | 9.4508 | 3.1408 | 0.25 |
| 130 | 8.76761 | 4.5008 | 0.34 |
| 131 | 9.30019 | 3.49957 | 0.27 |
| 132 | 9.74093 | 2.6518 | 0.21 |
| 133 | 8.87389 | 4.5508 | 0.34 |
| 134 | 9.22795 | 3.86311 | 0.30 |
| 135 | 8.74537 | 4.8749 | 0.36 |
| 136 | 9.32442 | 3.3593 | 0.26 |
| 137 | 9.48207 | 3.0338 | 0.24 |
| 138 | 10.0571 | 1.40622 | 0.12 |
| 139 | 9.26252 | 3.25564 | 0.26 |
| 140 | 9.51548 | 2.68775 | 0.22 |
| 141 | 9.55998 | 2.74758 | 0.22 |
| 142 | 9.00498 | 4.3797 | 0.33 |
| 143 | 9.37774 | 2.6151 | 0.22 |
| 144 | 8.92774 | 3.52365 | 0.28 |
| 145 | 10.444 | 0.00010444 | 0.00 |
| 146 | 9.47947 | 2.21036 | 0.19 |
| 147 | 9.58444 | 1.80705 | 0.16 |
| 148 | 9.11456 | 3.11625 | 0.25 |
| 149 | 8.92381 | 3.82513 | 0.30 |
| 150 | 9.22972 | 2.70707 | 0.23 |
| 151 | 9.4419 | 1.9488 | 0.17 |
| 152 | 8.24932 | 5.12108 | 0.38 |
| 153 | 9.49297 | 1.76229 | 0.16 |
| 154 | 9.17673 | 2.53747 | 0.22 |
| 155 | 8.94328 | 2.8128 | 0.24 |
| 156 | 9.01915 | 2.51543 | 0.22 |
| 157 | 8.86135 | 3.01097 | 0.25 |
| 158 | 9.33048 | 1.55166 | 0.14 |
| 159 | 10.0411 | 0.00010041 | 0.00 |
| 160 | 8.98053 | 2.42793 | 0.21 |
| 161 | 9.09563 | 1.95033 | 0.18 |
| 162 | 9.9487 | 9.9487E-05 | 0.00 |
| 163 | 9.16063 | 1.82728 | 0.17 |
| 164 | 8.68032 | 3.12729 | 0.26 |
| 165 | 8.68929 | 2.79547 | 0.24 |
| 166 | 8.01457 | 4.39155 | 0.35 |
| 167 | 8.66902 | 2.93433 | 0.25 |
| 168 | 8.61305 | 3.05848 | 0.26 |
| 169 | 8.39253 | 3.417 | 0.29 |
| 170 | 9.71492 | 9.7149E-05 | 0.00 |
| 171 | 8.20255 | 3.54946 | 0.30 |
| 172 | 9.6521 | 9.6521E-05 | 0.00 |
| 173 | 8.06305 | 4.17772 | 0.34 |
| 174 | 8.70505 | 2.15751 | 0.20 |
| 175 | 8.92491 | 1.42276 | 0.14 |
| 176 | 8.92402 | 1.3766 | 0.13 |
| 177 | 8.57618 | 2.34161 | 0.21 |
| 178 | 8.47712 | 2.45998 | 0.22 |
| 179 | 8.75488 | 1.77022 | 0.17 |
| 180 | 8.71052 | 1.67524 | 0.16 |
| 181 | 9.42244 | 9.4224E-05 | 0.00 |
| 182 | 8.77608 | 1.46077 | 0.14 |
| 183 | 9.33696 | 9.337E-05 | 0.00 |
| 184 | 7.70309 | 4.22185 | 0.35 |
| 185 | 8.62718 | 1.64298 | 0.16 |
| 186 | 8.48783 | 1.92437 | 0.18 |
| 187 | 8.7365 | 1.16718 | 0.12 |
| 188 | 9.24541 | 9.2454E-05 | 0.00 |
| 189 | 8.44306 | 1.97781 | 0.19 |
| 190 | 9.18107 | 9.1811E-05 | 0.00 |
| 191 | 9.15401 | 9.154E-05 | 0.00 |
| 192 | 8.2744 | 1.98622 | 0.19 |
| 193 | 8.35208 | 1.89721 | 0.19 |
| 194 | 9.0887 | 9.0887E-05 | 0.00 |
| 195 | 8.47795 | 1.4682 | 0.15 |
| 196 | 9.07141 | 9.0714E-05 | 0.00 |
| 197 | 7.6307 | 3.91063 | 0.34 |
| 198 | 8.15723 | 2.23978 | 0.22 |
| 199 | 8.2008 | 2.00479 | 0.20 |
| 200 | 8.13946 | 2.2848 | 0.22 |
