# Supplementary material for: The quantitative genetics of gene expression in Mimulus guttatus
Source: PLoS Genet. 2024 Apr 11;20(4):e1011072. doi: 10.1371/journal.pgen.1011072 (PMC11060551; doi:10.1371/journal.pgen.1011072)
Supplement: S2 Fig — (PDF) [file pgen.1011072.s012.pdf]

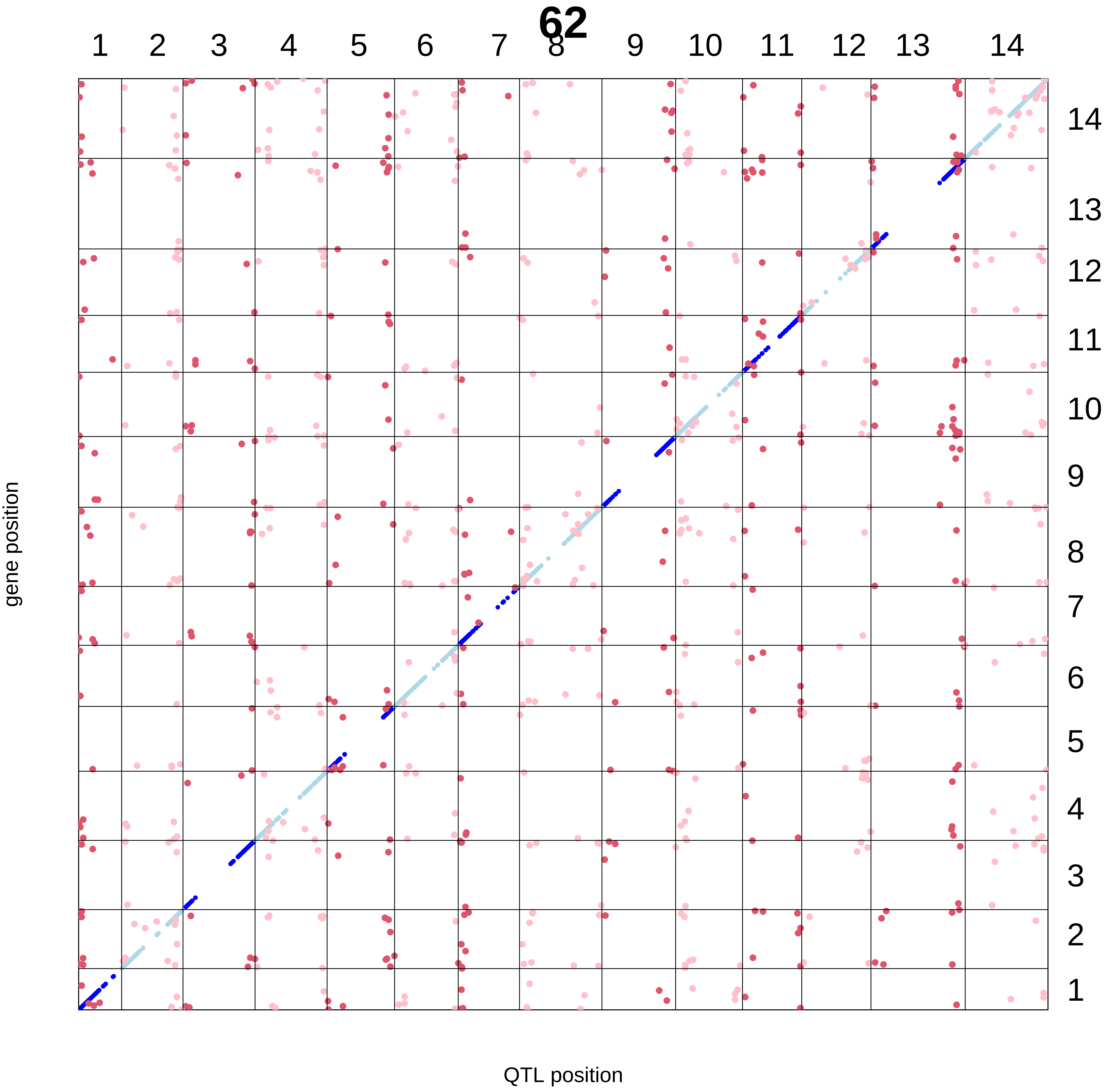
62
1
2
3
4
5
6
7
8
9
10
11
12
13
14
gene position
QTL position

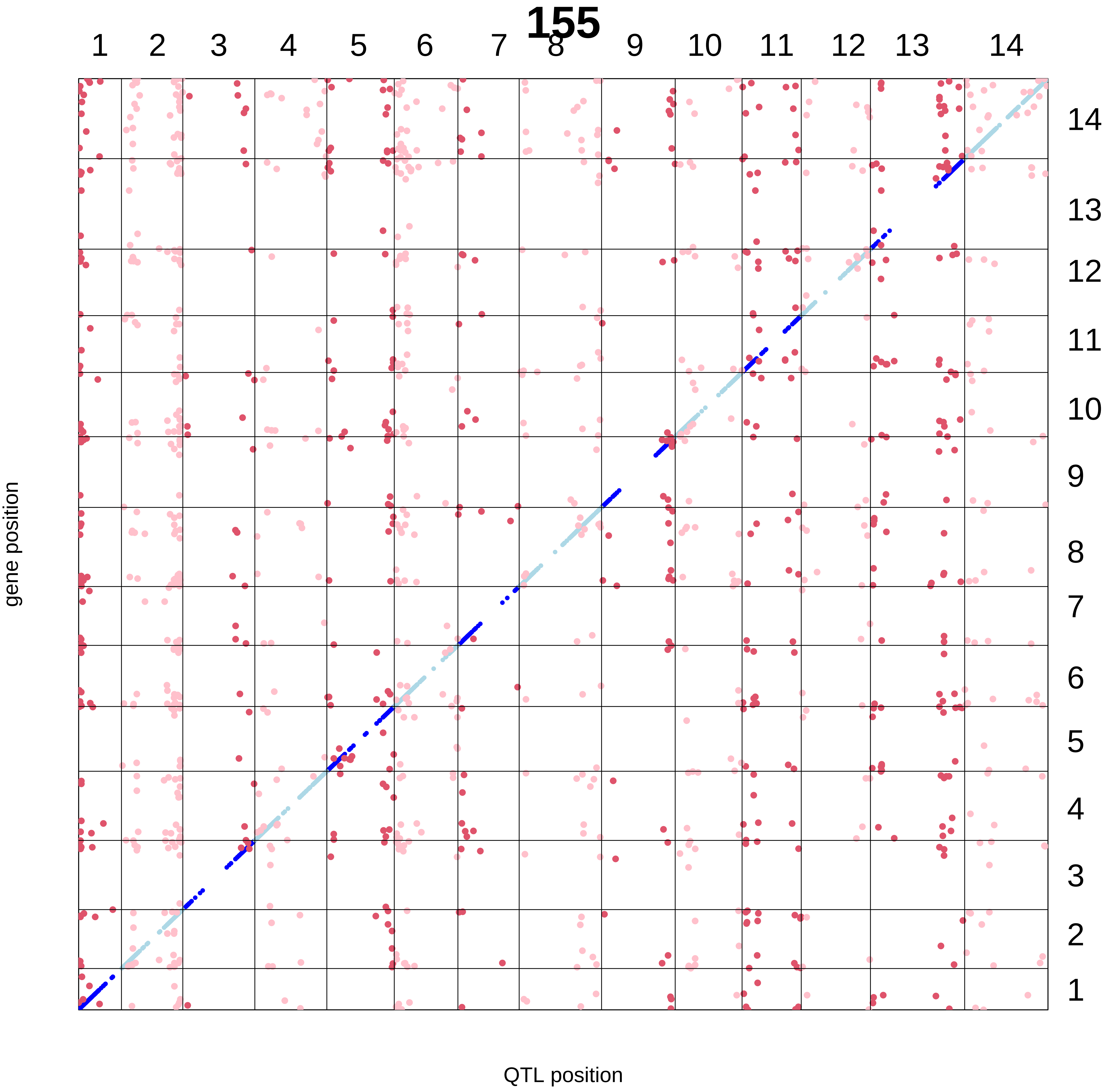
155
1
2
3
4
5
6
7
8
9
10
11
12
13
14
gene position
QTL position

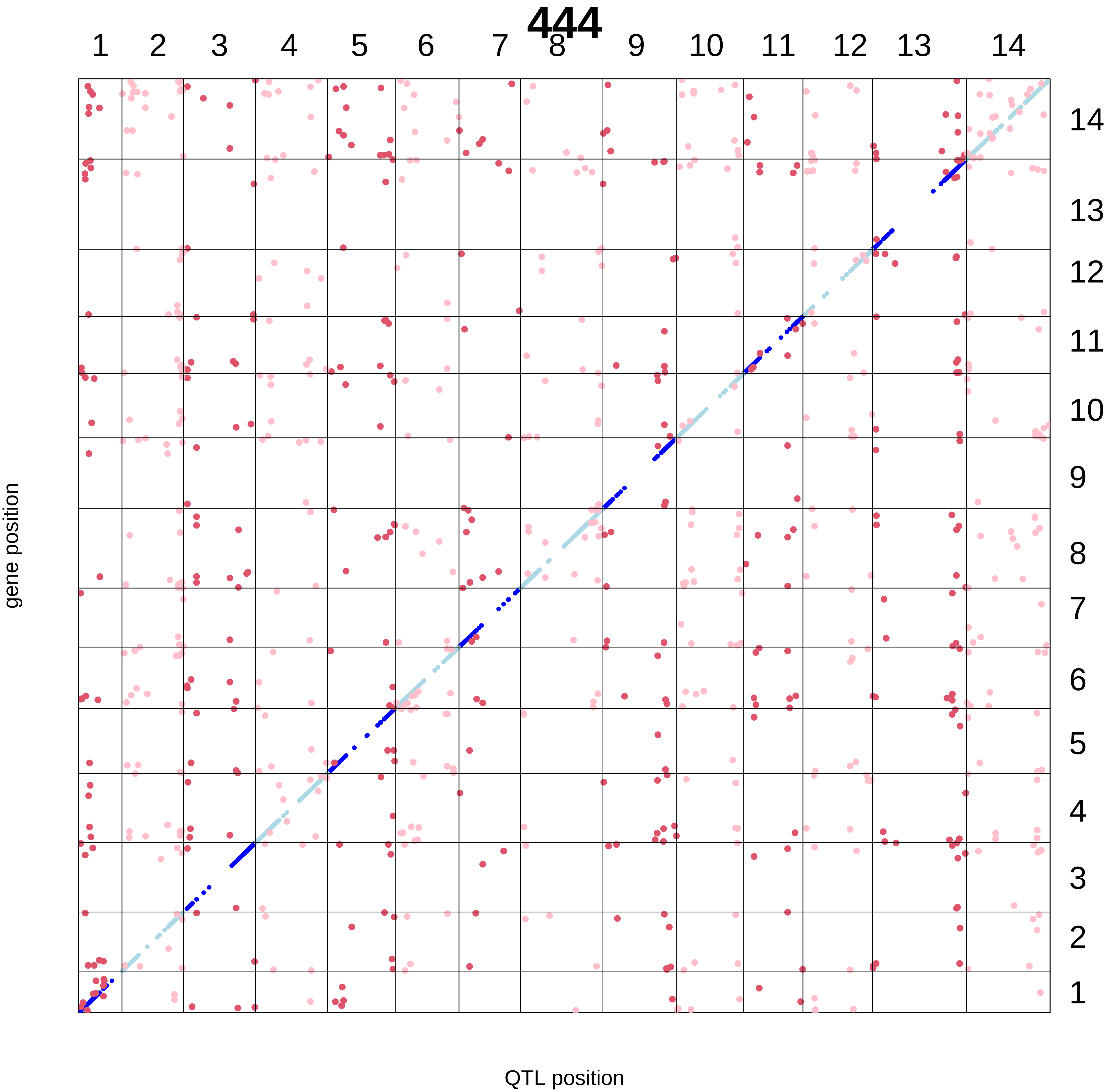
444
1 2 3 4 5 6 7 8 9 10 11 12 13 14
14 13 12 11 10 9 8 7 6 5 4 3 2 1
gene position
QTL position

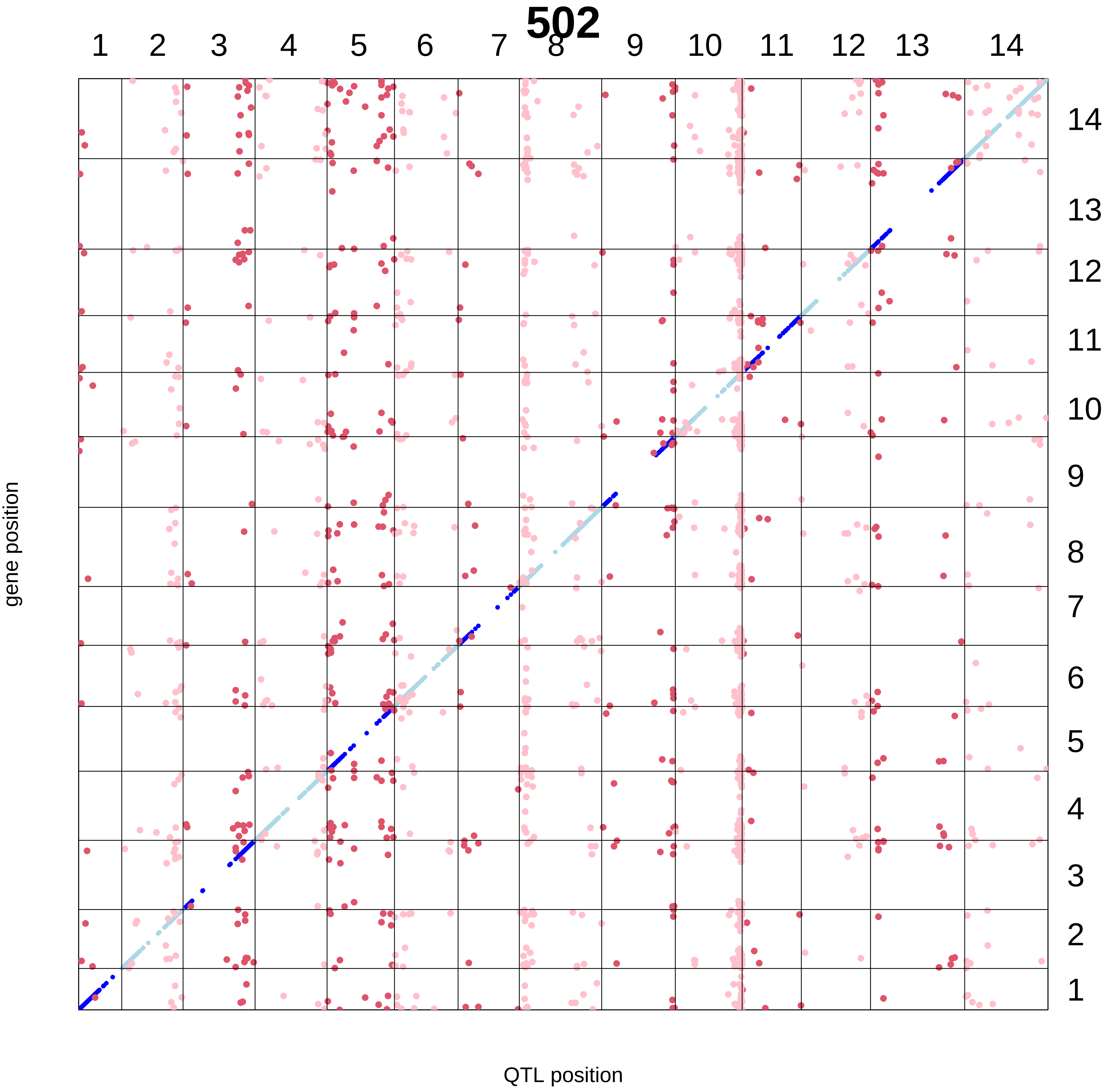

502
1
2
3
4
5
6
7
8
9
10
11
12
13
14
QTL position
gene position

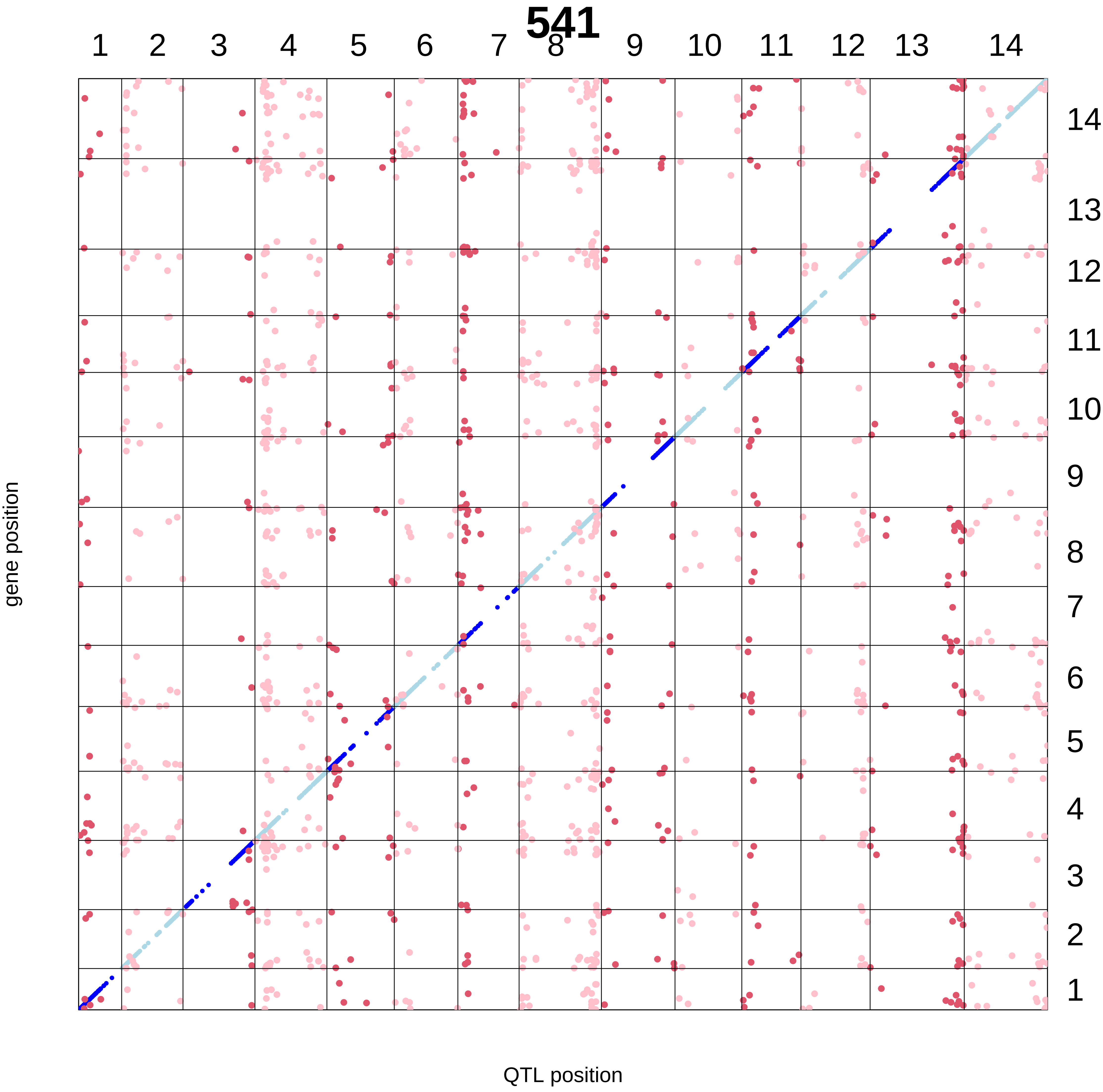

541
1
2
3
4
5
6
7
8
9
10
11
12
13
14
gene position
QTL position

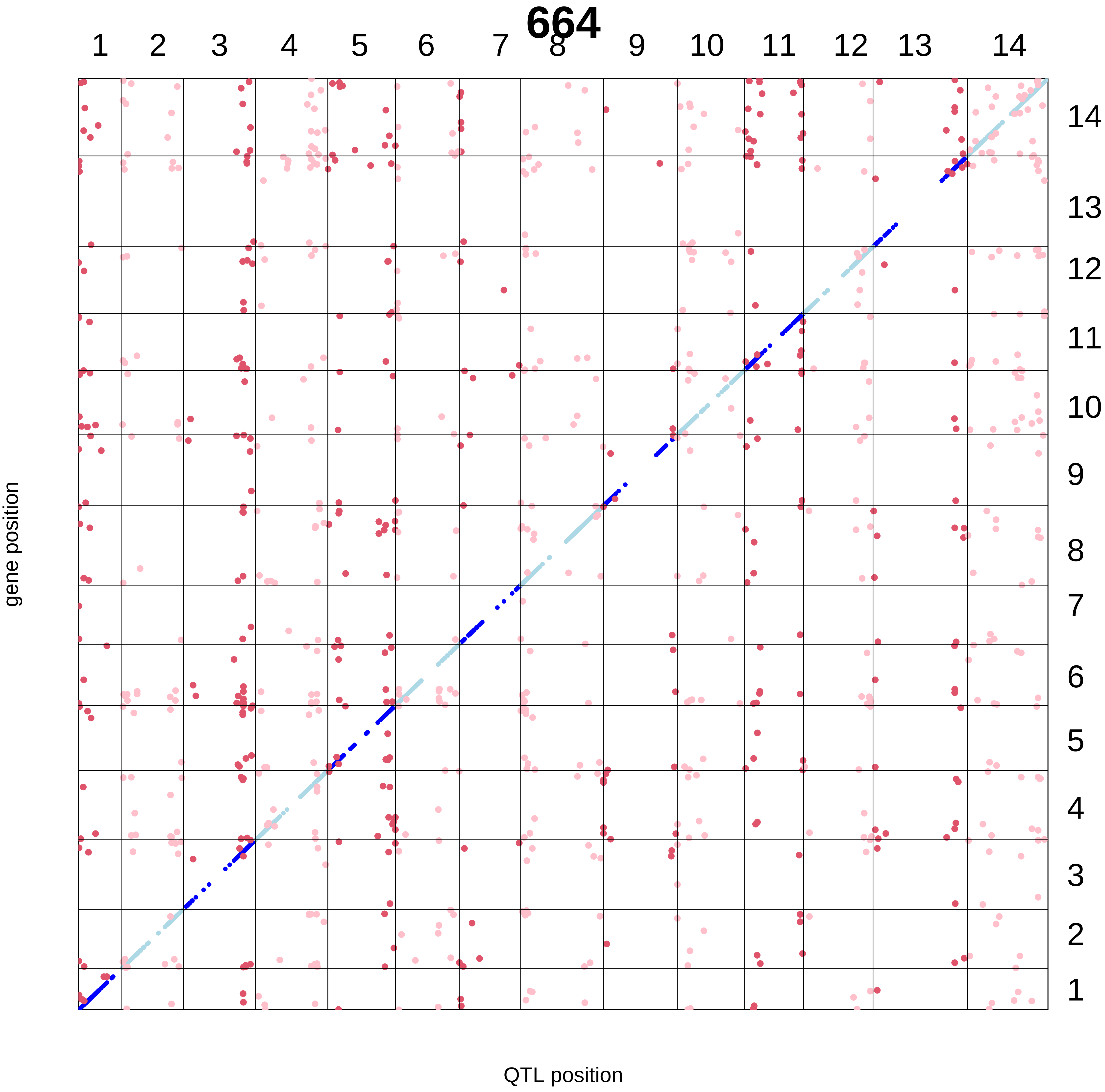
664
1
2
3
4
5
6
7
8
9
10
11
12
13
14
gene position
QTL position

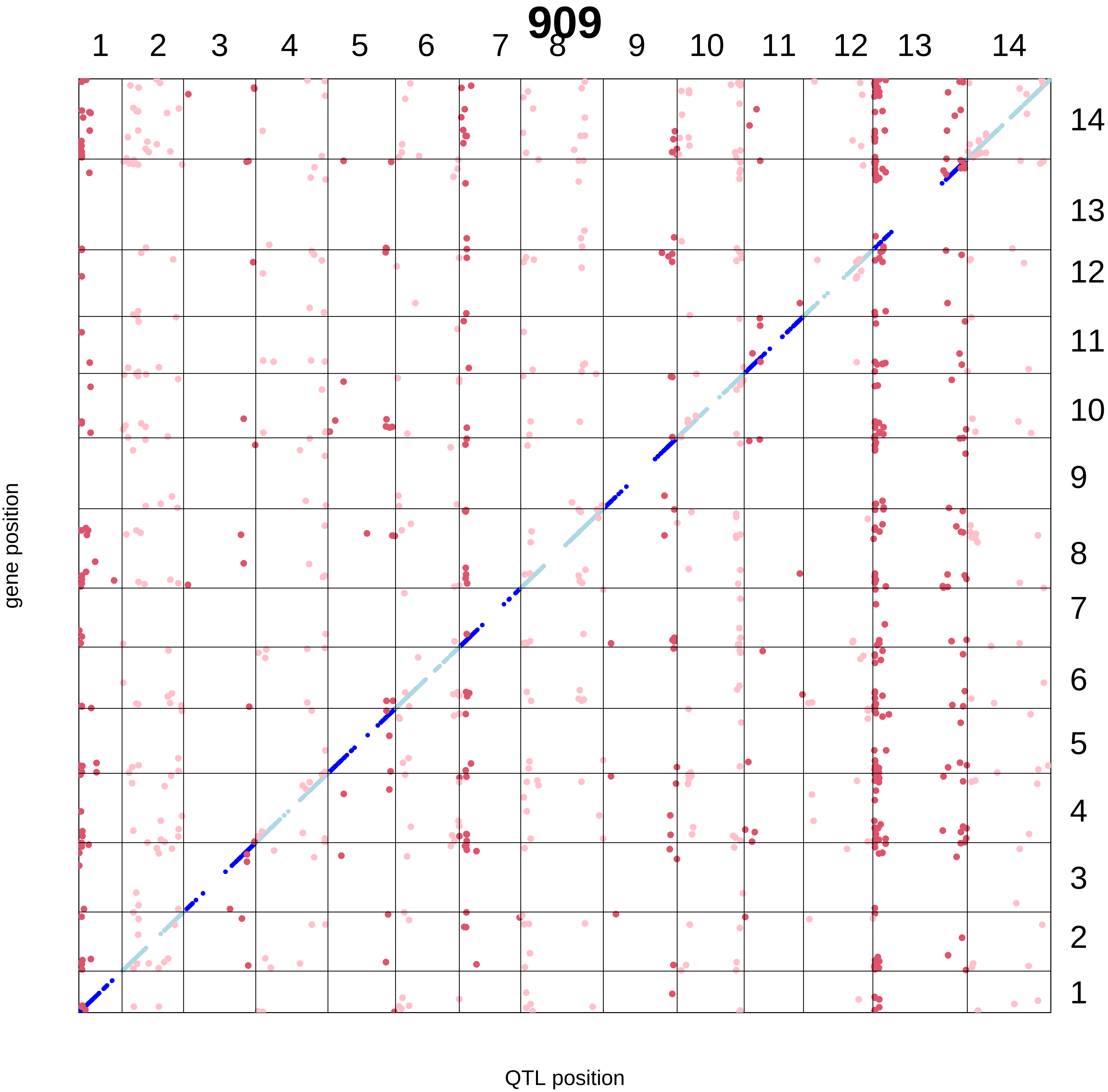

909
1
2
3
4
5
6
7
8
9
10
11
12
13
14
gene position
QTL position

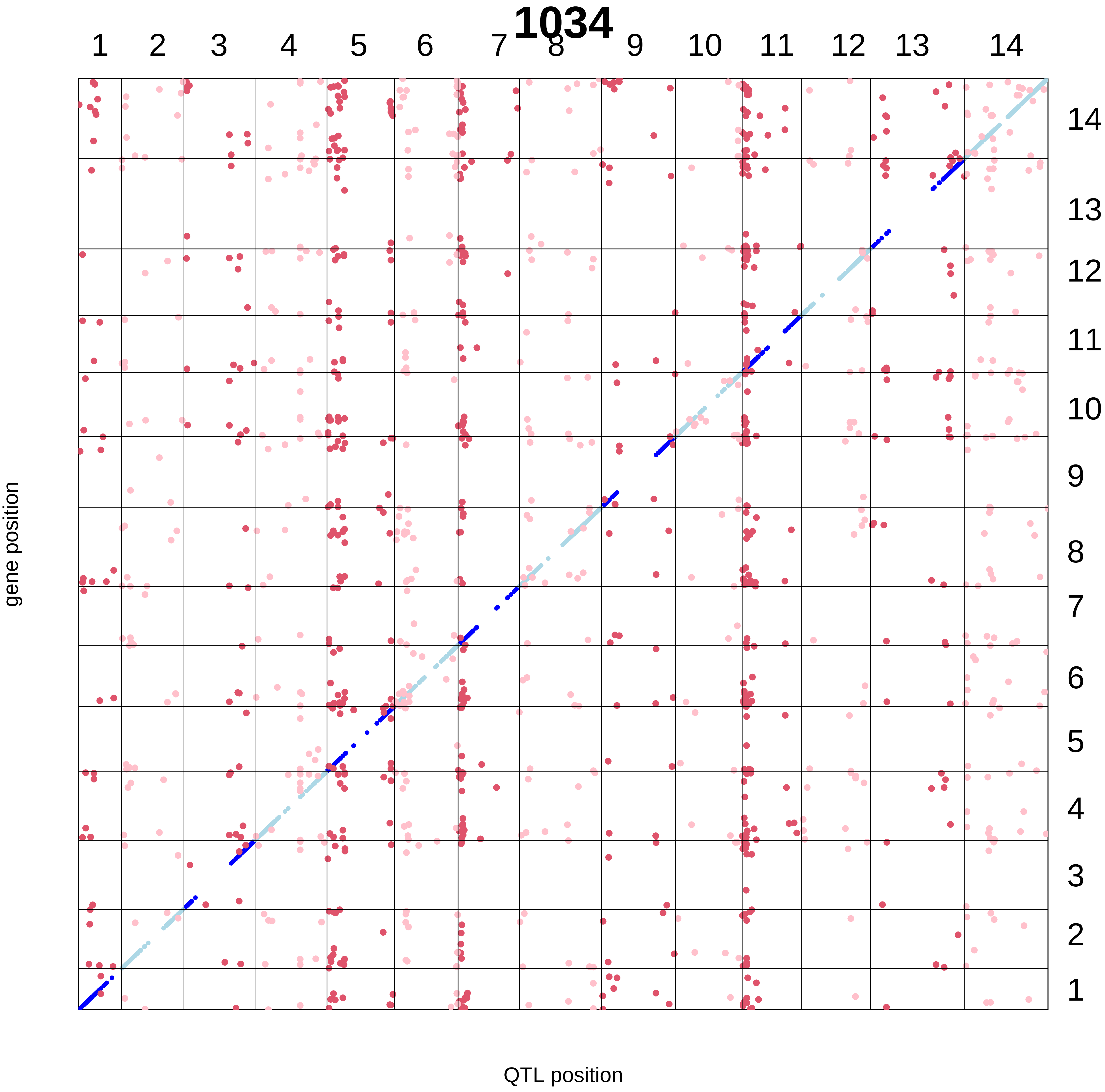
1034
1
2
3
4
5
6
7
8
9
10
11
12
13
14
gene position
QTL position

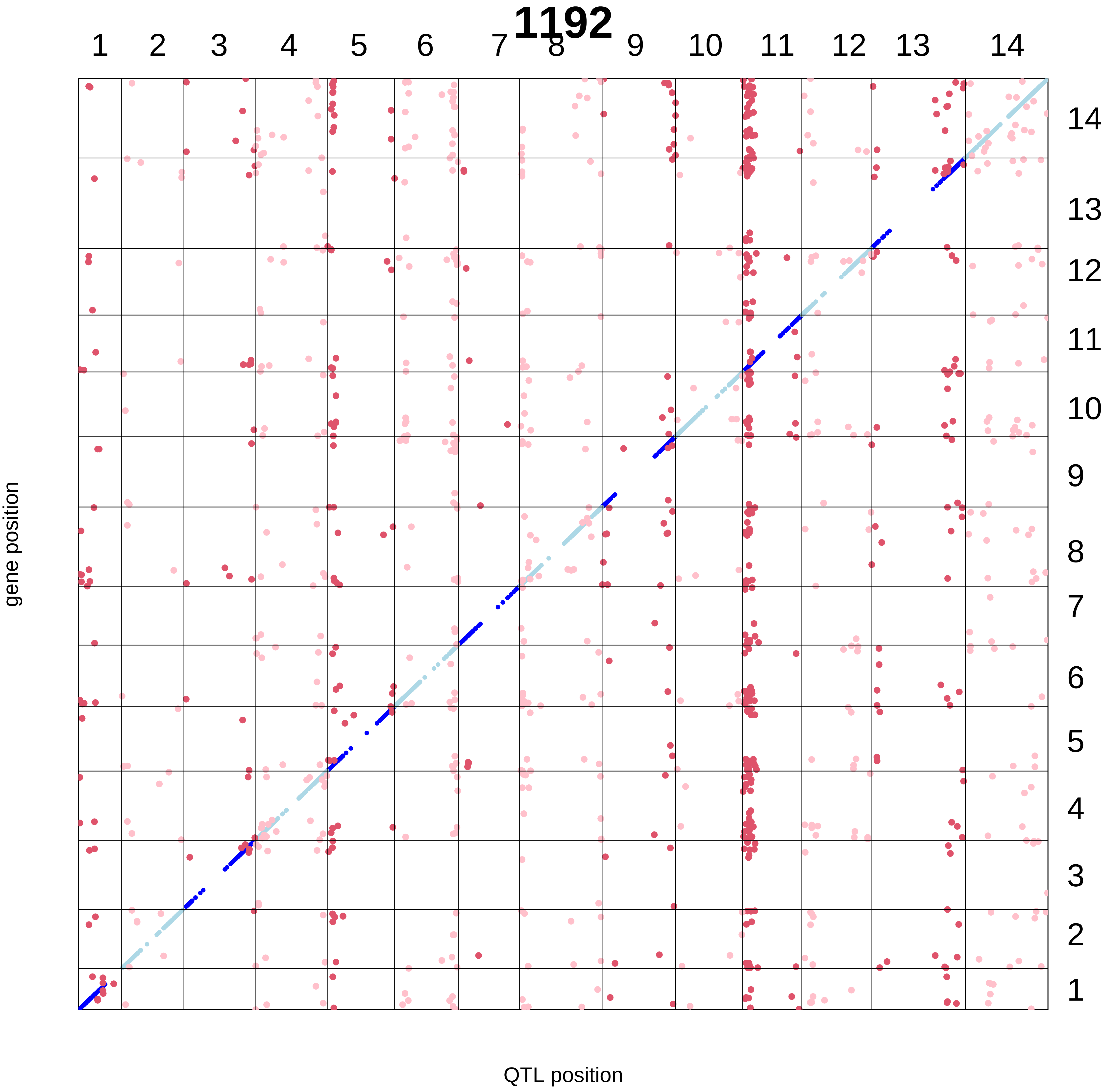
1192
1 2 3 4 5 6 7 8 9 10 11 12 13 14
gene position
QTL position
14 13 12 11 10 9 8 7 6 5 4 3 2 1
