# Supplementary material for: The quantitative genetics of gene expression in Mimulus guttatus
Source: PLoS Genet. 2024 Apr 11;20(4):e1011072. doi: 10.1371/journal.pgen.1011072 (PMC11060551; doi:10.1371/journal.pgen.1011072)
Supplement: S3 Fig — We divided all gene regions into quartiles (x-axis) and averaged the Log CPM across genes within each region. (PDF) [file pgen.1011072.s013.pdf]

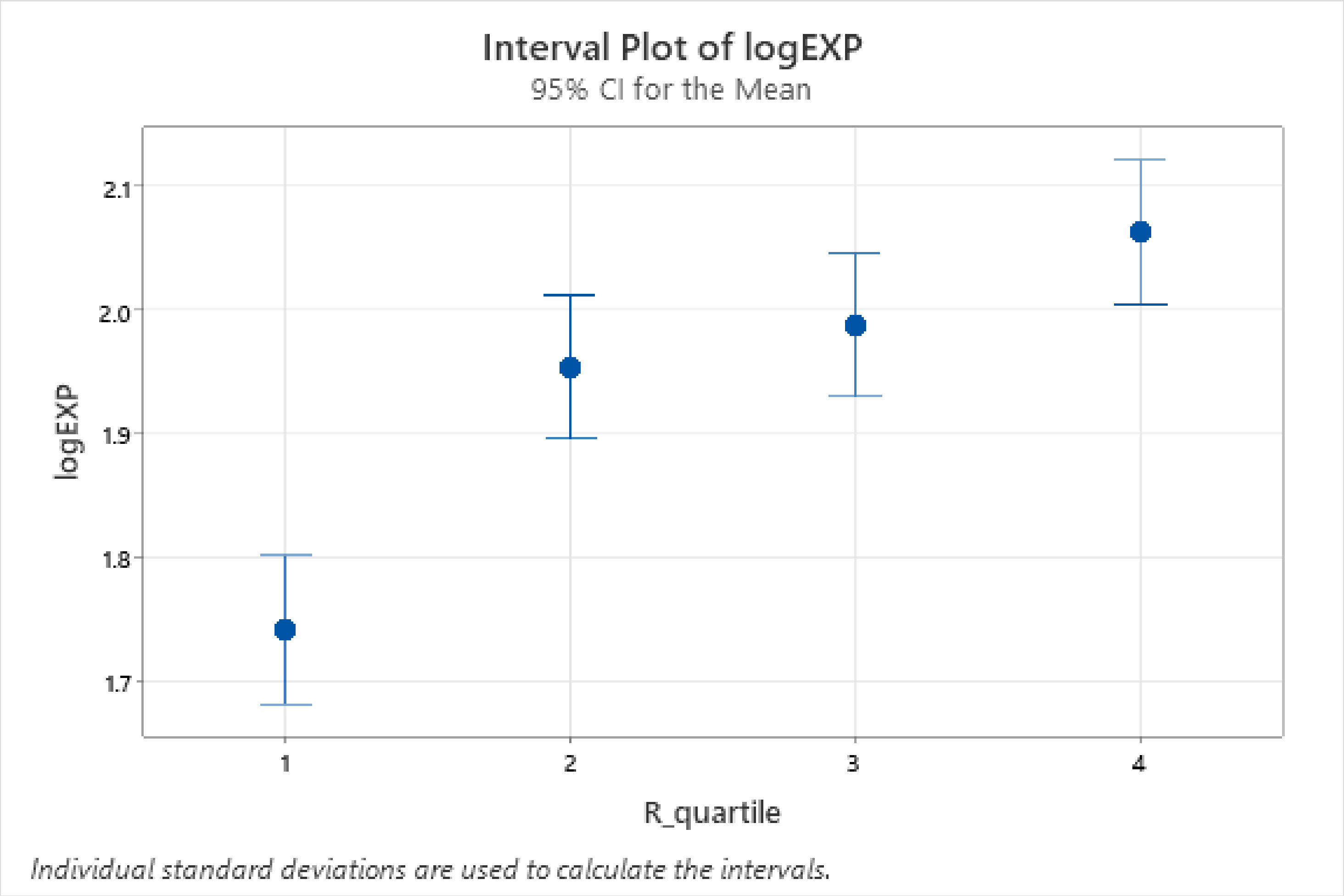

Supplementary figure 3. The rate of recombination within a chromosomal region affects average gene expression. We divided all gene regions into quartiles (x-axis) and averaged the Log CPM (count per million) across genes within each region.
