# Supplementary material for: The quantitative genetics of gene expression in Mimulus guttatus
Source: PLoS Genet. 2024 Apr 11;20(4):e1011072. doi: 10.1371/journal.pgen.1011072 (PMC11060551; doi:10.1371/journal.pgen.1011072)
Supplement: S4 Fig — (PDF) [file pgen.1011072.s014.pdf]

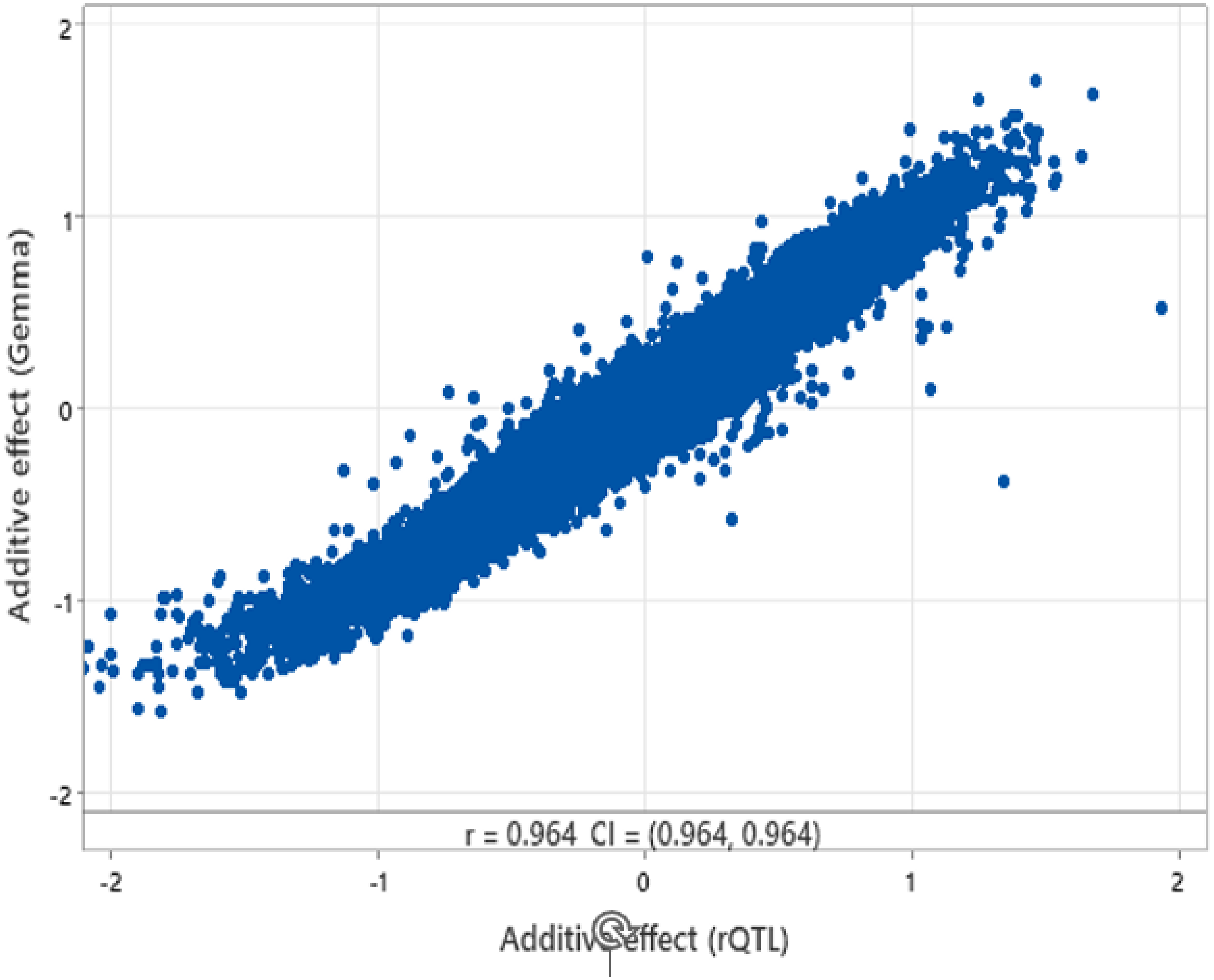

Supplementary figure 4. The correlation of additive effect estimates between the Cross-specific (x-axis) and Combined (y-axis) analyses is 0.96.
