# Supplementary material for: The quantitative genetics of gene expression in Mimulus guttatus
Source: PLoS Genet. 2024 Apr 11;20(4):e1011072. doi: 10.1371/journal.pgen.1011072 (PMC11060551; doi:10.1371/journal.pgen.1011072)
Supplement: S5 Fig — (PDF) [file pgen.1011072.s015.pdf]

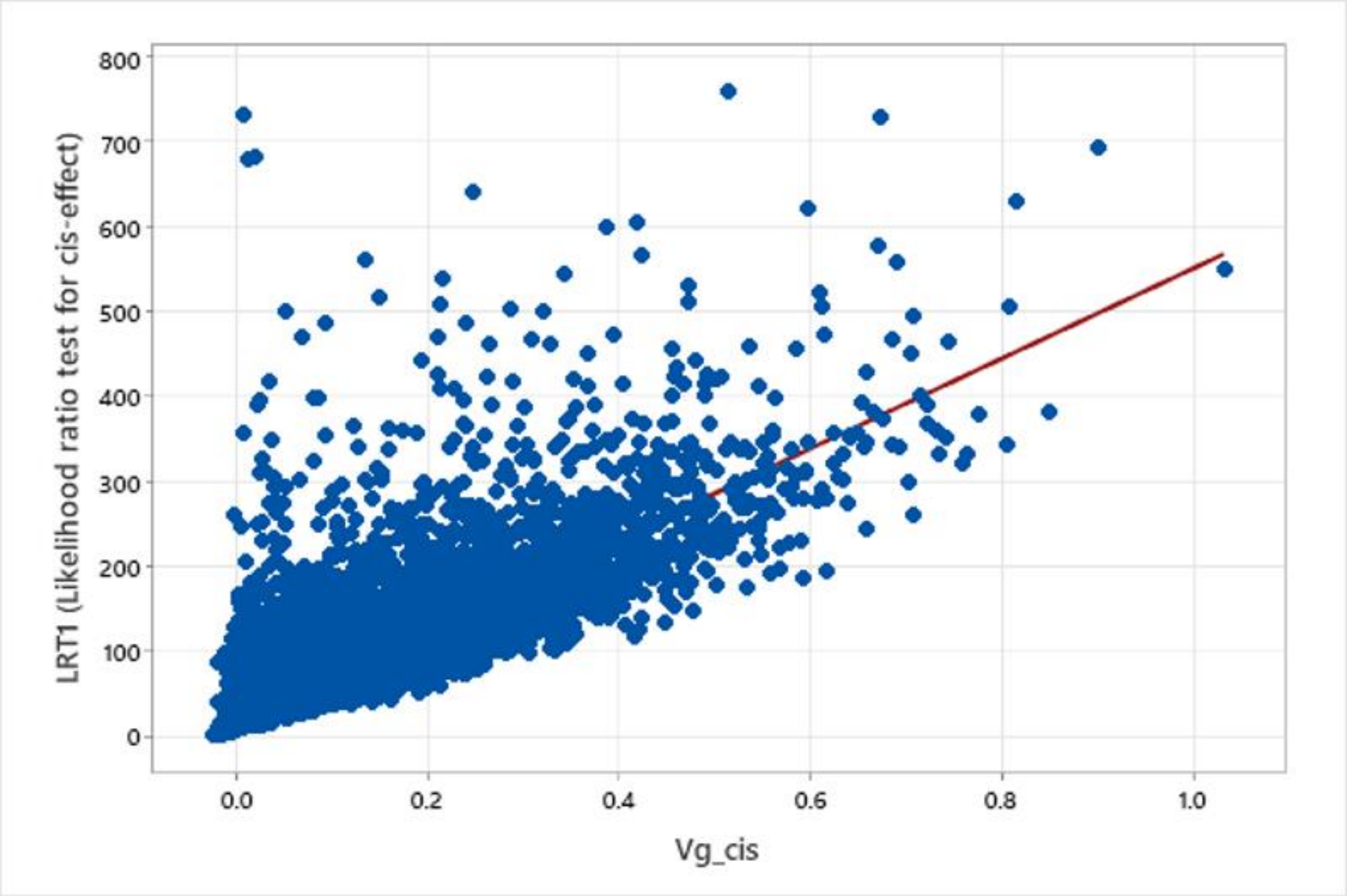

Supplemental figure 5. The strength of evidence for a cis eQTL (LRT1) is positively correlated with $V_{g(cis)}$.
