# Supplementary material for: The quantitative genetics of gene expression in Mimulus guttatus
Source: PLoS Genet. 2024 Apr 11;20(4):e1011072. doi: 10.1371/journal.pgen.1011072 (PMC11060551; doi:10.1371/journal.pgen.1011072)
Supplement: S6 Fig — Mean expression (measured as CPM on a log scale) is a strong positive predictor of test significance (left) and a strong negative effector of VE (right). (PDF) [file pgen.1011072.s016.pdf]

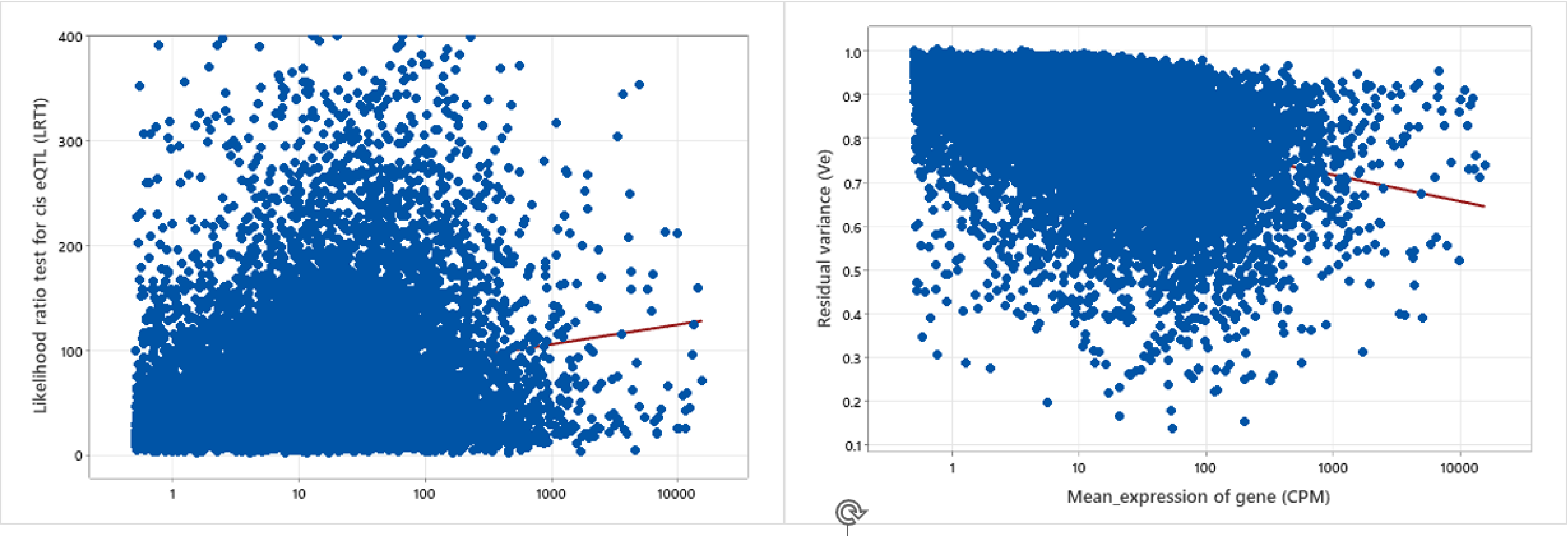

Supplemental figure 6. Mean expression (measured as CPM on a log scale) is a strong positive predictor of test significance (left) and a strong negative effector of V_E (right).
