# Supplementary material for: The quantitative genetics of gene expression in Mimulus guttatus
Source: PLoS Genet. 2024 Apr 11;20(4):e1011072. doi: 10.1371/journal.pgen.1011072 (PMC11060551; doi:10.1371/journal.pgen.1011072)
Supplement: S7 Fig — GG (left) and CE (right) each exhibit distributions with a roughly equal mixture of positive and negative values. (PDF) [file pgen.1011072.s017.pdf]

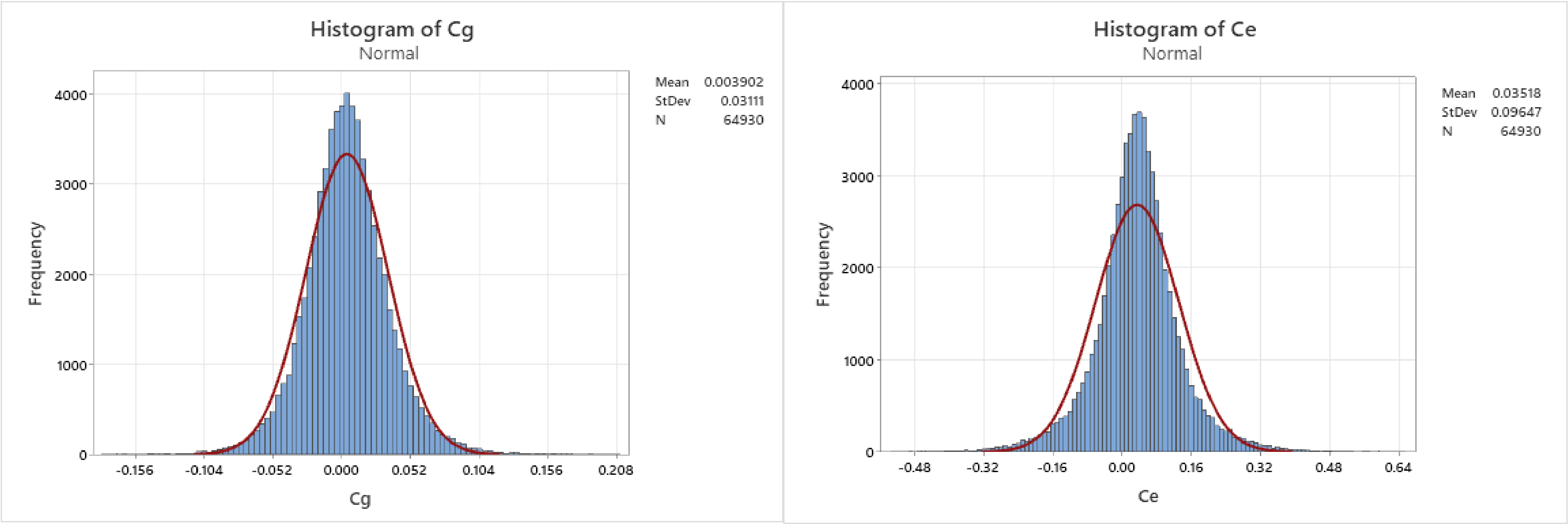

Supplementary figure 7. G_G (left) and C_E (right) each exhibit distributions with a roughly equal mixture of positive and negative values.
