# Supplementary material for: The quantitative genetics of gene expression in Mimulus guttatus
Source: PLoS Genet. 2024 Apr 11;20(4):e1011072. doi: 10.1371/journal.pgen.1011072 (PMC11060551; doi:10.1371/journal.pgen.1011072)
Supplement: S8 Fig — (PDF) [file pgen.1011072.s018.pdf]

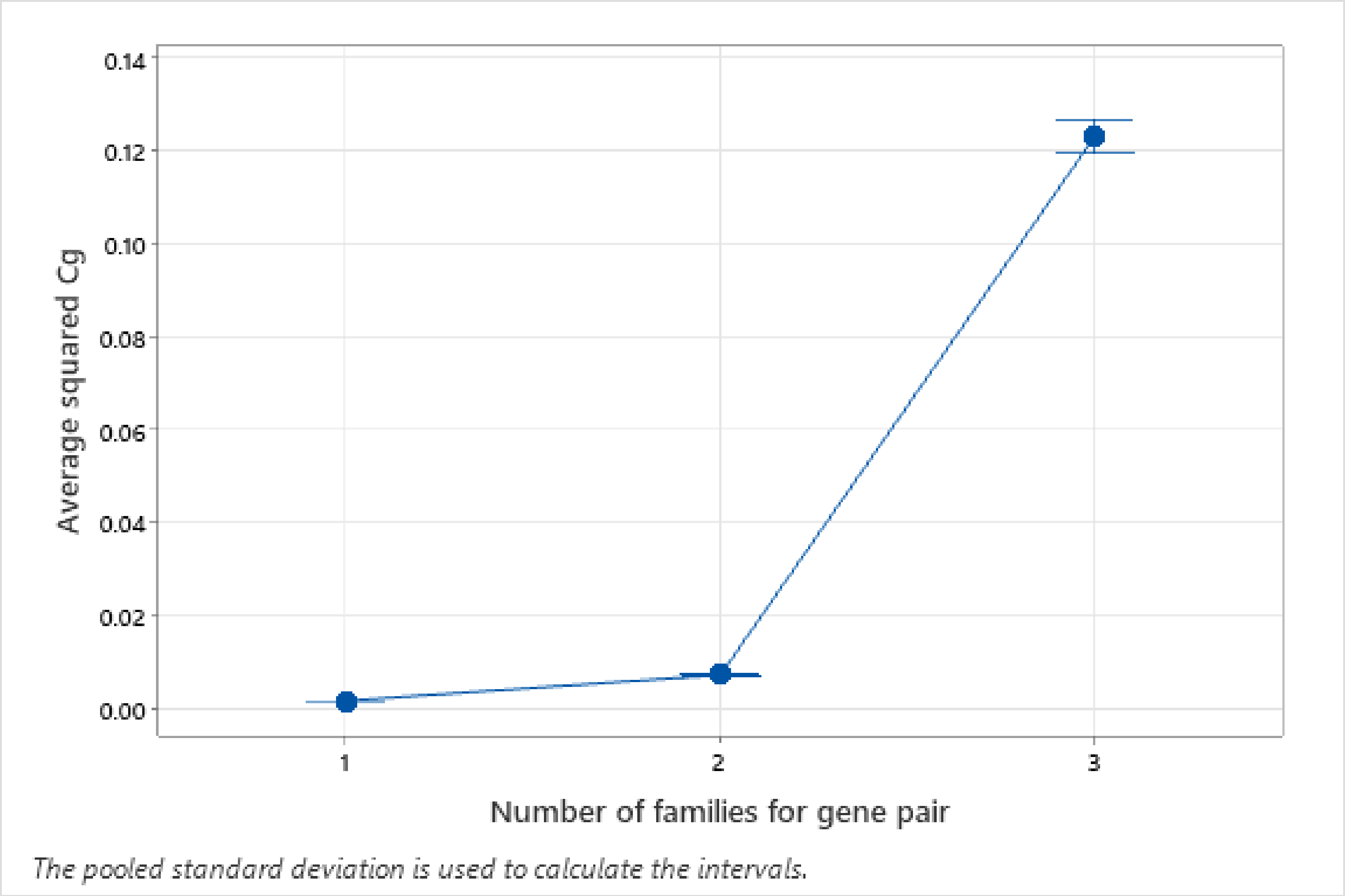


**Supplementary figure 8. The magnitude of the genetic covariance (mean $C_G^2$) increases with the number of families that segregate a trans eQTL.**
