# Supplementary material for: The quantitative genetics of gene expression in Mimulus guttatus
Source: PLoS Genet. 2024 Apr 11;20(4):e1011072. doi: 10.1371/journal.pgen.1011072 (PMC11060551; doi:10.1371/journal.pgen.1011072)
Supplement: S9 Fig — The actual mean of simulations (8.77) is slightly below the null distribution predicted value of 9 for a chi-square distribution with 9 df. (PDF) [file pgen.1011072.s019.pdf]

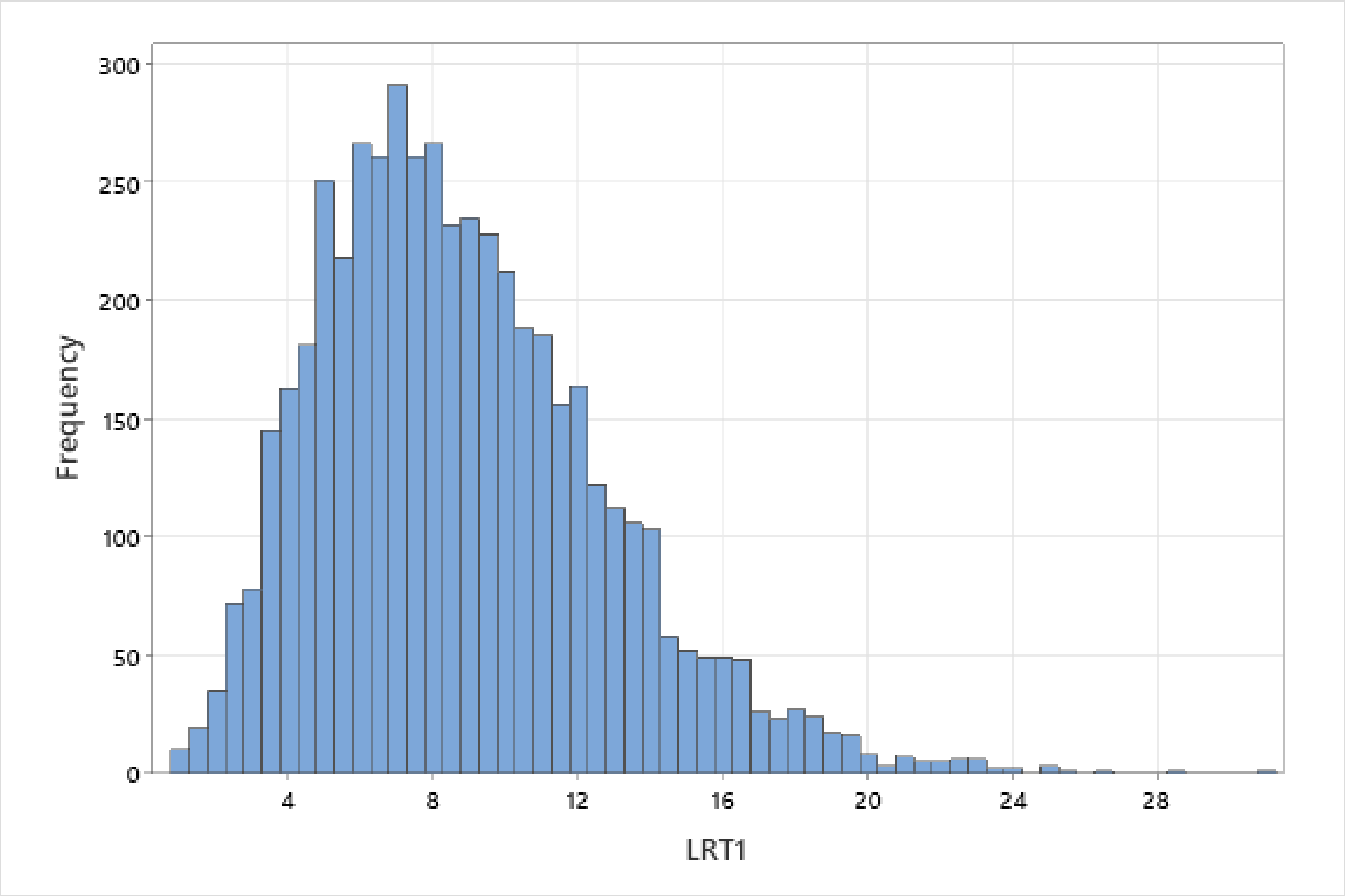

Supplementary figure 9. The distribution of LRT1 (the test for an additive eQTL) across 5000 simulations when there is no eQTL. The actual mean of simulations (8.77) is slightly below the null distribution predicted value of 9 for a chi-square distribution with 9 df.
