# Supplementary material for: The quantitative genetics of gene expression in Mimulus guttatus
Source: PLoS Genet. 2024 Apr 11;20(4):e1011072. doi: 10.1371/journal.pgen.1011072 (PMC11060551; doi:10.1371/journal.pgen.1011072)
Supplement: S1 File — (GZ) [file pgen.1011072.s020.gz › Supplemental_File_1/Key_to_programs.docx]

The programs listed below were written in python 3 tested on Ubuntu linux. Command line arguments are enclosed [*] following python program names.

**Program Set 1**

The first set of programs identify the genes annotated in the reference line (IM767), determine their locations in the nine other genomes, and then make the “diploid transcriptomes” used for read mapping. This set also includes the programs that analyze sequence differences (SNPs and indels) given Mummer alignments of each alternative assembly to IM767.

The first input for subsequent alignments, "IM767v1.genes.txt", was created by running **genes.in.767.annotation.py**. The Liftoff software (see Methods) produced "767_lift_to_[genom].gff3" for each alternative assembly [genom]. Next, we ran **liftoff_genes_to_767positions.py** [genom] **cross_specific.gffs.py** [genom] and to produce the alignment files. Next **make.gene.fasta.from.gff3.py** [genom] creates the gene specific mapping reference for Salmon (see Methods) with two sequences for each gene (one specific to each parental genome). Finally, **analyze.salmon.transcript_fa.py** [genom.filelist.txt] [genom] processes the Salmon output files to extract the quantitative mappings of reads to each allele of each gene in each plant. The gene ordering with Liftoff transcriptomes was also useful to assemble scaffolds into the chromosomes. IM62 was already a chromosome level assembly but the other eight alternatives needed to have scaffolds stitched (ordered and oriented) into chromosomes. This was done running **contig_location_stats.py** [genom] and **make.psuedo.builds.py** [genom] on each genom.

To score differences in the nucleotide sequences among our assemblies, we used Mummer 3.0 [1] and SVMU [2] with the following commands applied to each alternative genome (cross):

nucmer --threads 28 --prefix [cross]_TO_767 767.fa [cross].fa

dnadiff --prefix [ cross]_TO_767dnadiff3 -d [cross]_TO_767.delta

svmu [cross]_TO_767.delta 767.fa [cross].fa snp_mode=h [cross]_TO_767_lastz.txt [cross]_TO_767_svmu_out

Taking the outputs from Mummer and SVMU for each cross ([cross]_TO_IM767dnadiff2.1coords, [cross]_TO_IM767dnadiff2.snps), we ran **read.mummer1.py** [chrom] and **read.mummer.v3.py** [chrom] for each chromosome. Given the specified window size (window = 1000), these program first identify all the correctly aligned positions between each line and 767. Next, they count the number of positions that are a gap in 767, a gap in the alternative line, or an aligned SNP. Report counts as alt line, gap 767, gap alt, aligned calls that are snps. Next, **Mummer_indel_analysis.py** takes these outputs and considers all genes, aggregating polymorphism information for within and around each gene. Finally, **Mummer_indel_bygene.py** takes calculates the polymorphism statistics (pi and U) to predict cis eQTL significance.

**Program Set 2**

Set 2 are programs from the GOOGA pipeline [3], revised for the current application.

These programs extract genetically informative markers from all genes, impose the HMM for estimation using scipy optimization tools to find the MLE for parameters. After obtaining the posterior genotype probabilities at each marker, we establish a genotype matrix (including all genes between markers) by interpolation. This matrix is then used to calculate the relatedness matrix as well as the input files for linear mixed model fits in gemma and the single marker analyses in rQTL.

The mapping data for each plant at each gene is scored for parentage (whether reads map to 767, or the alternative genome, or both. This produces a putative genotype call.

We first run **s1.py** [cross] on each cross (family) to identify the genetically informative genes in this cross. This program also outputs the genes with suitable for allele specific expression analysis in this family. Next, we run **s2.py** [cross] to make preliminary genotype calls based on read depth and allele frequency thresholds. Given preliminary calls, we run **s3.py** [cross] to identify aberrant markers (because they exhibit excessive disagreement with neighboring markers) and plants with poor data. Next, we implement the HMM on each chromosome of each cross across individuals. In the program **s4.py** [cross], we hold recombination rate to 5 cM / mb to obtain initial genotyping error rates (e1 = probability that a homozygous plants emits a heterozygous genotype call, b1 = probability that a heterozygous plants emits the reference homozygote call, b2 = probability that a heterozygous plants emits the alternative homozygote call). These are chromosome wide estimates (within a family), used as initial values for subsequent programs allow marker specific rates. The next program, **s5.py** [cross], is the primary estimation tool. Progressing marker by marker along chromosomes, this program estimates recombination rates (between adjacent markers) and error rates (e1,b1,b2 for each marker) using the HMM forward algorithm for to calculate the likelihood given (current) parameter values. The cycling over markers within each chromosome is repeated until the likelihood converges on the maximum. At convergence, the program outputs the MLE for recombination rates and marker specific gentoyping error rates. These are input to **s6.py** [cross], which applies the forward-backward algorithm to estimate genotype posterior probabilities. These are output both as numbers to each possible genotype and as an assignment to the most probable genotype at each marker for each plant.

We use the genotype posterior probabilites from s6.py to make input files for the Cross Specific and Combined analyses. We run **make.rQTL.f2geno.file.py** [cross] to make the input files for rQTL. **gene.by.marker.by.cross.py** identifies the marker closest to each gene within each cross, which is the input to **genotype.per.gene.py** [cross] which interpolates the genotype at each gene to be analyzed for expression. To make the relatedness matrix, the program **run.Relatedness.by.plant.py** produces many scripts applying the program **Relatedness.by.plant.py** to each plant. We concatenate these outputs to make “RelationshipMatrix.txt”

**Program Set 3**

This set of programs applies the linear mixed model of Gemma to the expression level of each gene. First, we create the phenotype files using **BoxCoxExpression.py** [gene]. The program **Make.cis.fixedeffects.py** [gene] reads the cis genotype for each plant and creates the “covariate file” of Gemma with the cis genotype expressed as 9 parameters (additive model) or 18 parameters (dominance model). We standardize all traits to unit variance and store in [gene].Pheno.txt. Next, **run.gemma.3models.py** launches many calls to:

gemma -g IG.txt -p [gene].Pheno.txt -n 1 -c cohorts.txt -k RelationshipMatrix.txt -lmm -o [gene].x1

gemma -g IG.txt -p [gene].Pheno.txt -n 1 -c [geno].cis.txt -k RelationshipMatrix.txt -lmm -o [gene].cis

gemma -g IG.txt -p [gene].Pheno.txt -n 1 -c [geno].cis_d.txt -k RelationshipMatrix.txt -lmm -o [gene].cis_d

where Gemma [4] is the program fitting the linear mixed model. Here, IG.txt is the input file for an uninformative SNP (one with no effect on phenotype). The -lmm module is typically used for association mapping on biallelic SNPs. Here, we are only interested in the estimated variance components and the estimates of the cis genotype (which is included as a fixed effect in the covariates file).

To distill the results from the output files of gemma, we run **Analyze.lmm_3models.py** [gene] to extract the maximum log-likelihood under each model, fixed effects, and variance component estimates. Finally, we calculate the LRT for both the additive and dominance models, calculate test p-values (assuming chi-square with 9 or 18 degrees of freedom), and then use R to calculate the False Discovery Rate for each test on each gene:

library(data.table)

dat<-read.table("pval_add.txt",header=FALSE)

names(dat)<-c("p")

Qval = p.adjust(dat$p,method="fdr")

fwrite(list(Qval), file = "add.Qval.txt")

To estimate the number of functionally distinct alleles at each cis eQTL, the program

**create.partition.10alleles.py** was run to make the file “**partitions_10.txt**”, which lists all possible partitions of 10 distinct alleles. Given the ranked list of allelic effects for each gene, **cis.allele.number.v2.py** outputs all the possible allelic configurations for each gene as [geneid].possible.alleles.txt. Next, **Run.multiple.allele.pipeline.v2.py** launches many calls to **Make.cis.multiple_alleles.v2.py** [geneid]. This program tests all 511 configurations for each gene in gemma with the output files preserved to extract the log-likelihood values. After all cases are considered, **Best.model.each_allele_number.py** [geneid] identifies the partition with the highest likelihood for each possible allele number and the reports best overall model based on LRT tests applied to each increase in allele number. Finally, **read.allele_numbers.cis.py** calculates heterozygosity based on the chosen model and outputs the allelic configuration. Essentially the same set of operations were applied to trans eQTLs using a parallel set of programs: **trans.allele.number.py**, **Run.multiple.allele.pipeline.trans.py**, **Make.trans.multiple_alleles.py**, **Best.trans.model.allele_number.py**, and **read.allele_numbers.trans.py**. To produce the summaries for allele specific expression analyses, we ran **run.ASE.py** which creates scripts for many calls to **Allele.Specific.Expression.py** [geneid]. We aligned these outputs (the frequency of alt-allele in heterozygote expression and whether allele-specific expression is significant) for each gene to the allelic effect estimates from Gemma to produce Fig 3 in main text.

**Program Set 4**

To perform principal components analysis, on the transcriptome, we ran **Standardized.expression.matrix.py** to produce a matrix of standardized transcript levels (each gene has mean zero and variance 1) with all genes in one file. With R, we ran:

library(MASS)

library(dplyr)

library(data.table)

dat<-read.table("std.matrix.txt",header=TRUE)

my_pca <- prcomp(dat, scale = TRUE, center = TRUE, retx = T)

sink('PC_var_explained.txt')

summary(my_pca)

sink()

write.matrix(my_pca$rotation, file="pc_loadings.csv",sep=",")

write.matrix(my_pca$x, file="pc_scores.csv",sep=",")

The output “pc_loadings.csv” has each PC as a column, each gene as a row (after header). The output “pc_scores.csv” has each PC as a column and the loadings for each plant as a row (after header). Next, we ran **predict_pcscores.py** to confirm “pc_scores.csv” contains the correct numbers. Finally, **pcscores_for_gemma.py** converts the data into gemma formatted phenotype files (a separate text file for each PC) and **pull_loadings.py** makes a separate file for each gene with the loadings for that gene for each PC.

**Program Set 5**

This set includes the simulation programs used to test different variance component estimation methods. For each parameter set, we first specify Ve and the genetic parameters Vg_cis and Vg_trans in the header of the program **Data.simulation.normal.py**. The many scripts to run replicate simulations are produced by **run.estimation.simulations.py**. Each replicate is initiated by randomly selecting a genes from full list of tested genes. The first program run in each replicate is

**Data.simulation.normal.py** [geneid], which reads the gemma formatted covariates file for this gene (which contains the cis genotype for all plants at this gene). Given the cis genotype and the full relatedness matrix, the program simulates and assigns a cis effect, a trans effect, and an environmental deviation to each plant. This is output as a Gemma formatted phenotype file. Given these outputs, and the relatedness matrix and covariates file for the gene, we Gemma to fit Models 0, 1, and 2 exactly as with the real data (as described above for Program Set 3). Given the Gemma outputs, we run **lmm_sim_results.py** [geneid]. This program extracts the ML for each model, all the parameter estimates, calculates LRT1 and LRT2 and performs the tests. Here, we also retain the HE estimates although they are not used in the analysis of the actual data.

Cited:

1. Kurtz S, Phillippy A, Delcher AL, Smoot M, Shumway M, Antonescu C, et al. Versatile and open software for comparing large genomes. Genome Biology. 2004;5(2):R12. doi: 10.1186/gb-2004-5-2-r12.

2. Chakraborty M, Emerson JJ, Macdonald SJ, Long AD. Structural variants exhibit widespread allelic heterogeneity and shape variation in complex traits. Nature Communications. 2019;10(1):4872. doi: 10.1038/s41467-019-12884-1.

3. Flagel LE, Blackman BK, Fishman L, Monnahan PJ, Sweigart A, Kelly JK. GOOGA: A platform to synthesize mapping experiments and identify genomic structural diversity. PLOS Computational Biology. 2019;15(4):e1006949. doi: 10.1371/journal.pcbi.1006949.

4. Zhou X, Stephens M. Genome-wide efficient mixed-model analysis for association studies. Nat Genet. 2012;44(7):821-4. Epub 2012/06/19. doi: 10.1038/ng.2310. PubMed PMID: 22706312; PubMed Central PMCID: PMCPMC3386377.
