# Supplementary material for: The quantitative genetics of gene expression in Mimulus guttatus
Source: PLoS Genet. 2024 Apr 11;20(4):e1011072. doi: 10.1371/journal.pgen.1011072 (PMC11060551; doi:10.1371/journal.pgen.1011072)
Supplement: S2 File — (PDF) [file pgen.1011072.s021.pdf]

HMW DNA extraction plants

Materials needed

- Mortar and pestle, at -20ºC
- Liquid N_2 tank
- (2) β-mercaptoethanol and fume hood access (5th floor)
- Cold resistant gloves
- Corning 70 µm cell strainer
- Cooled Falcon centrifuge, 8th floor
- 6 Falcons per sample

Tissue collection

Collect 5 gr leaf tissue in one falcon tube per sample.

Day 1

Setup

1. Make NIB and CLB solutions.
2. Prepare liquid N_2, ice box. Take cell strainers, Triton.
3. Add BME to NIB in fume hood.
4. Per sample: 1 labelled falcon tube with 50 mL 0ºC NIB, 1 labelled empty tube and 1 tubes with 2.5 ml NIB + 0.25 µl Triton, on ice.
5. Turn on water bath at 74ºC and 8th floor centrifuge.
6. Setup paper towel on bench to contain mess.

Extraction

1. Grind tissue in liquid N_2 (30 sec grinding after evaporation x3). Add powder to NIB tube.
2. Strain with Corning 70 µm cell strainer into prepared cold falcon tube (swirl with pack of blue tip to help along, through away gunk when too much).
3. Strain again with same cell strainer in falcon tube with Triton.
4. Centrifuge at 4ºC for 10 min @4000 RPM.
5. Remove supernatant. Add 10 ml Carlson Lysis Buffer + 25 µl BME to each pellet. Incubate at 74ºC for 2 h, swirl every 30 min.
6. Cool to room temp. Label 2 tubes per sample.
7. Add 10 ml chloroform (0.75% ethanol). Invert gently to mix.
8. Centrifuge at 4ºC for 10 min @4000 RPM. Label new Falcon.
9. Transfer aqueous phase (non-green) to new tube. Use 1 ml pipette. Leave some behind.
10. Add chloroform to 20 ml mark (about 10 ml - equal volume) so all samples are balanced in weight.
11. Repeat: spin, label new Falcon and transfer aqueous phase to new tube.
12. Add 1 ml 3M NaOAc and mix gently.
13. Add 11 ml (equal volume) isopropanol (by eye on falcon tube). Gently mix.
14. Precipitate at -20ºC overnight (should not freeze).

Day 2

Setup

1. Turn on centrifuge at 4ºC.
2. Get ice and cool down 70% ethanol.

Extraction

1. Centrifuge for 30 min at 4ºC.
2. Discard supernatant. Add 40 ml 70% ethanol.
3. Centrifuge for 30 min at 4ºC.
4. Discard supernatant.
5. Leave tubes at angle on tissue to air dry in fume hood.
6. Add 100 µl TE buffer per sample. Leave at 4ºC for 2 hr.
7. Transfer to 1.5 ml eppendorfs.

Recipes

Table 1: NIB Nucleus Isolation Buffer. KBnF: Kelly Bench next to Fridge, KCd: Kelly Cupboard down, KCu: Kelly Cupboard up, KF: Kelly fridge, FH: Fume Hood 5th floor, LH: Lena Hileman lab.

In 500 ml bottle	**unit**	**5 gr sample**	**2 samples + 10 %**	**4 samples + 10 %**
**spermine**^{KF}	g	0.01	0.022	0.045
**EDTA**^{KCd}	g	0.19	0.41	0.82
**KCl**^{KCu}	g	0.38	0.82	1.64
**sucrose**^{KCu}	g	8.56	18.83	37.66
**spermidine**^{KCd}	µl	31.25	68.75	137.5
**Tris pH 9.5**^{KBnF}	ml	0.5	1.1	2.2
**BME**^{FH} **(before use)**	ml	0.05	0.11	0.22
**ddH$_2$O**^{LH} Mark level before adding everything in bottle	add to ml	50	110	220

Table 2: CLB Carlson Lysis Buffer.

In 50 ml Falcon	**unit**	**5 gr sample**	**2 samples**	**4 samples**
**Tris pH 9.5**^{KBnF}	ml	1	2	4
**2% CTAB powder**^{KCu}	g	0.2	0.4	0.8
**NaCl**^{KCd}	g	0.82	1.64	3.28
**1% PEG 6000**^{KF}	ml	0.6	1.2	2.4
**EDTA**^{KCd}	g	0.07	0.15	0.3
**ddH$_2$O**^{LH}	add to ml	10	20	40

<u>Sodium Acetate (NaOAc$^{\text{6th floor cupboard}}$)</u>
6.15 gr in 25 ml H_2O (in Falcon)
