# Supplementary material for: The quantitative genetics of gene expression in Mimulus guttatus
Source: PLoS Genet. 2024 Apr 11;20(4):e1011072. doi: 10.1371/journal.pgen.1011072 (PMC11060551; doi:10.1371/journal.pgen.1011072)
Supplement: S1 Fig — We calculated π for each chromosome, and then averaged these 14 values to obtain the mean and standard error (the latter used to calculate the 95% CI). (PDF) [file pgen.1011072.s011.pdf]

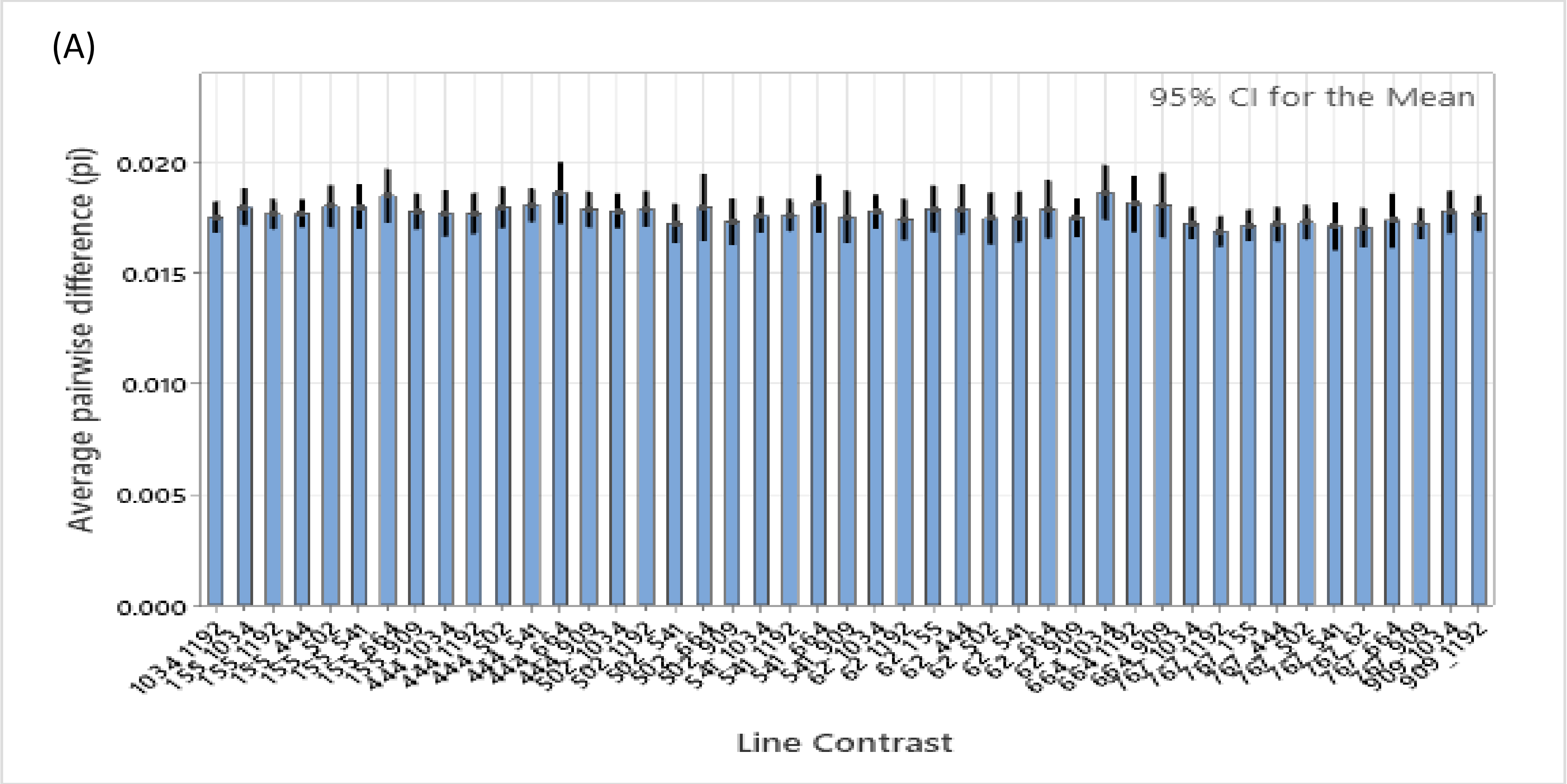

Supplementary figure 1. (A) The fraction of nucleotide positions that differ (p) within genes is reported between each pair of lines. We calculated π for each chromosome, and then average these 14 values to obtain the mean and standard error (the latter used to calculate the 95% CI). (B) The genetic maps obtained for each family.
