# Supplementary material for: The quantitative genetics of gene expression in Mimulus guttatus
Source: PLoS Genet. 2024 Apr 11;20(4):e1011072. doi: 10.1371/journal.pgen.1011072 (PMC11060551; doi:10.1371/journal.pgen.1011072)
Supplement: Attachment — Submitted filename: Response_letter_Veltsos_Kelly_2_26_2024.pdf [file pgen.1011072.s022.pdf]

Dear Drs Lasky and Dyer,

I am submitting a revised version of PGENETICS-D-23-01298 (The quantitative genetics of gene expression in Mimulus guttatus). The initial draft of this paper received very positive reviews that contained many useful suggestions for revisions. We have adopted most of these suggestions and revised the ms in response to nearly all. The point-by-point responses are given below.

Sincerely,

John Kelly

Lawrence, KS, 2/26/2024

Reviewer comments and responses (**in bold**).

Dear Dr. Kelly,
I have now received three reviews of your manuscript. Overall, the reviewers appreciated the study for the depth and scope of quantitative genetics findings on gene expression. The study is well designed and the dataset and analyses are rich.
However, perhaps reflecting the complexity of the topic, there are many points of clarification that the reviewers emphasized. Next, as one reviewer noted, the connection between your findings and underlying evolutionary processes is particularly interesting. There was some discussion of this in the manuscript, but the reviewer and I felt it could be sharpened and strengthened. Additionally, reviewers noted that the provided code appeared to be missing important analyses.
I look forward to receiving a revised version of the manuscript.
Sincerely,
Jesse Lasky

Response: Regarding the connection evolutionary processes, see detailed comments to suggestions to all three referees. Regarding the code, we are much more explicit in the revised draft. At the very top of the Methods (pg 19 of revised draft), we have added this text:

"All software developed by others, including standard bioinformatic tools such as Salmon [70] and Gemma [23], are reported as used. Most of our analyses were completed using custom programs written in Python (v3.7). These programs are provided in Supplemental File 1 along with "Key_to_programs.docx" which describes their use."

Importantly, we have added two key references for the standard tools to the revised draft. The revised methods also make several additional pointers for particular analyses within Key_to_programs. Between the main text method and code and documentation in Supplemental File 1, I do not think that any code is missing for this revised draft.

Reviewer #1: This manuscript investigates genetic variation in gene expression in a population of Mimulus guttatus. It uses a multi-parental mapping population to avoid some of the pitfalls of GWAS-based approaches and more accurately estimate properties of gene expression variation. The manuscript shows that almost every gene has a cis-eQTL, many with multiple alleles segregating in the population. While there were detectable trans-eQTLs for many genes, they still did not explain most of the trans-expression variation, and they had stronger dominance and were at lower allele frequencies than cis-eQTL. In addition, the manuscript demonstrates how trans-eQTLs can

contribute to coexpression and shows that mapping expression PCs can detect eQTLs.

This manuscript's results fill in large gaps in our basic understanding of the genetics of gene expression. There are lots of hypotheses about how cis- and trans- eQTLs might differ from each other in terms of dominance, selection, diversity, and effect size but, until now, we haven't had very much solid data on this from plant species. If anything, I think the manuscript undersells how important these findings will be for future work in the evolution of gene expression. The methods and approaches will also be very useful for future studies. In general, the manuscript is well-written and clear, although I have a few comments about places where additional explanation or writing might increase clarity, as well as some other suggestions to help the reader. I do not have any major concerns about the research itself.

Comments (in order of where they relate to the manuscript).

It would be helpful to define 'cis' regulation when it is first mentioned on line 64.
Response: Now defined in first sentence of Abstract

Fig 1C – I was a bit thrown off by the scale of pairwise relatedness being from 0-2 instead of 0-1. Could you briefly explain the measure of relatedness in the legend instead of just in the methods?
Response: We define relatedness in the standard way as twice the coefficient of conancestry (see Fig 1 legend, lines 708-715). Since the coefficient of conancestry ranges from 0 to 1, R ranges from 0 to 2. We have unpacked the logic for those unaccustomed to working with homozygous genotypes (see lines 162-180 of revised ms), emphasizing the maximum for R is 1 when everybody is outbred.

Line 151 – it wasn't clear to me what "the cis genotype" means here.
Response: We have rewritten that text and removed the term cis genotype because we do not use that phrase subsequently.

Line 185 – Could you add in either the number of genes with a cis-eQTL in the combined analysis or the percent of genes with cis eQTLs in the cross-specific analysis to make it easier to compare the two values?
Response: Done with lines 201-203 of revised. We also explain why the counts from the two analyses are not easily comparable.
Thinking of those who might skim this paper so they can cite the result "X% if genes have a cis-eQTL", could you be clearer about how this percent is out of the genes that were investigated, not all genes. It could be helpful to add in that this means that at least Y% of all genes have a cis-eQTL where Y is the number of genes with a mapped cis-eQTL divided by the total number of genes.
Response: Text added to line 196: "*These estimates refer only to measured genes, those expressed in leaf tissue and passing filters*."
Fig 2 – can you explain the differences in the colors of the dots?
Response: That is cis/trans with shades changing with chromosome – now explained in legend of that figure.
I couldn't figure the distance cut-off for whether a QTL was cis or trans. Can you include this info more prominently?
Response: We are more explicit on the Cross specific analysis in revision. See first paragraph of Methods D. Testing procedures.

Line 221 – could you replace "is usually" with a value for how often the allele that increases expression across

genotypes has higher allele-specific expression in heterozygotes?
Response: We added a new calculation to address this comment (line 248-251): "*This is illustrated by the subset of cases where the estimated additive effect of the alterative allele on expression is lower than -0.5 or greater than 0.5 (and we can be confident of correctly inferring up or down regulation). In these cases, allele frequency in the mRNA of heterozygotes deviates from 0.5 in the predicted direction in 99.6% of 7,841 tests*."

The analysis described on 232-236 took me a while to figure out – would it be possible to add a figure to explain how the dominance of a cis-eQTL affects allele-specific expression? One possibility would be plotting d on the x axis and the residual of the regression of the alt-allele frequency on a on the y axis? (this is not a requirement, and could possibly make things more confusing).
Response: We tried the figure but there are so many points overlaid, it is hard to see the positive correlation between the residual and d. Like the coefficient from the multiple regression, the correlation is highly significant. However, it is not large in magnitude owing to high estimation error in d. We have added some text to this section to try to make the meaning clearer (lines 257-263).
Line 288 – could you remind the readers of the average cis contribution to Vg here?
Response: Reminder of two thirds added.
Figure 4B be simplified by reporting the frequency of the most common allele, so for example columns [1,3,6] and [2,2,6] would be collapsed.
Response: We did not collapse because [1,3,6] and [2,2,6] are distinct cases with different H values. While the outcomes are slightly more complicated for two alleles than three, we think readers will be able to work through and understand 4B.
The reasoning of the paragraph starting on line 303 was a bit hard to follow. First, can you define H? Second, I think a bit more explanation about the comparison between the cross-specific and combined analysis would be helpful.
Response: H is definite slightly earlier (see line 276) and we have revised this bit to remind them what H denotes.
On line 369, it would be useful to have a bit more evidence that the pcQTLs overlap transeQTL hotspots, maybe in a supplemental figure or even reporting a value of how many pcQTLs are within 2 centimorgans of a hotspot or something.
Response: Text added on extent of overlap (lines 406-409) and clarified Appendix 2 as a more quantitative analysis of how pcQTLs absorb cis and trans eQTL effects.
Line 393 – the results about the relationship between sequence diversity at or around a gene and high Vg(cis) reminded me a bit of the results of Kremling et al. 2018 (https://doi.org/10.1038/nature25966), so it could be interesting to cite this and related papers here.
Response: Definitely relevant. Description with citation added to 2nd paragraph of Discussion.
Could you explain more about why different procedures were used for estimating read counts in the cross-specific and combined analyses? (line 654ish)
Response: Raw read counts were the same inputs to both pipelines, but the basic idea was a to contrast something standard and familiar to readers (the Cross specific analysis) with a quantitative genetic method that synthesizes the experiment as a whole. Despite many differences in detail, the effect estimates come out very similar (lines 204-213).

In the methods, could you add what programs or packages were used to fit the various models? I was a bit confused about why detailed code was given for the false discovery rate analysis (starting line 735) but not for other aspects of the statistical analyses or the simulations in Appendix 1.
Response: We open the revised methods with a statement on standard tools (cited below) and custom tools (described and included in Supplemental File 1). We added two important references on the standard tools to the Methods section in the main paper. The level of detail provide for the FDR in the original paper was just silly.

Now all command level detail has been moved into the "Key_to_programs" document of Supplemental File 1.

In the first set of null simulations, how was environmental variation in expression generated? (line 777)

Response: Using the generator in Numpy (see revised lines 849-851).

Reviewer #2: The study by Veltsos and Kelly assembles a truly stunning dataset comprising 10 F2 populations of the model ecological genetic species Mimulus gutatus, with 10 genetically diverse individuals collected from a single natural population each crossed to a common maternal line. This type of multi-parent recombinant population is perfectly suited for addressing classic quantitative genetic parameters such as the genetic variance and covariance, dominance, and genotype by environment interaction effects on traits. In the current study, the authors utilize these F2 individuals to estimate transcript abundances using RNASequencing – eQTL-- providing an unprecedented view into the genetic architecture of gene expression in plants. This work builds on, and substantially extends, a recent paper by this same group which used an association mapping approach to study the genetic architecture of gene expression using a broader sample of natural genotypes. The two studies strongly complement each other, and the authors do an excellent job of addressing similarities and differences in their results.

On the whole, I find this study remarkable and groundbreaking. It should serve as a template and benchmark for future eQTL studies, and deepens our understanding of the quantitative genetics of complex traits. The methods are well-executed, applying modern tools from quantitative genetics, genomics, and transcriptomics to classic questions in the evolution of complex traits.

Below are several suggestions to improve the manuscript. Most deal with presentation and interpretation.

Somewhat major points

The first paragraph (at Line 52) has too many un-expanded questions which lack context. I think this paragraph could be better utilized to make the case – in prose – that A. gene expression is a quantitative trait. B. there is a vast body of literature and theory on the evolution of quantitative traits. C. Many key processes in evolution can be studied by a modern, genome-enabled, perspective of A & B. In my view, this paper will be greatly appreciated by the quantitative genetics community but may be missed by the gene regulation and population genomics communities. Offering a gentle introduction to these latter communities would greatly increase the impact of this study.

Response: First paragraph has been revised along the lines suggested. We have 'unpacked' the questions by indicating how answering the questions addresses evolutionary hypotheses. The revised paragraph is longer but provides better motivation for the general audience. Numerous references have been added.

Line 64 – need a reference for cis-regulatory siting of expression variance. The following line "The priority of the cis-regulatory region…" also needs a reference, and a more general introduction to this important earlier observation would help the general reader.

Response: This text has been rewritten to explain the cis-regulatory locus (see lines 69-78 of revised ms) more clearly.

Line 138-139. Trying to get my head around this point. Wouldn't limited recombination, which limits mapping resolution, also make identifying the location of cis-eQTL harder? We know that the marker is linked to the causal variant, but with high LD it could be pretty far away even if technically in cis (linked)?

Response: Original text was confusing. We were speaking just about correctly inferring the genotype at the location of each gene. Limited recombination does limit mapping resolution, which is why we use the actual gene as the marker itself to test for cis effects on expression. See lines 142-147 of revised ms.

Line 157 – Support for the claim that the “crossing design produced the high variance in relatedness necessary for accurate estimation”? I don’t doubt this to be true, but this is written as a strong claim and I think some metric is warranted.
Response: We could report the variance in relatedness of each plant to all other plants but readers will have no familiarity with that number (to say high or low). As a substitute, we work through all the different sorts of contrasts per plant with differing values of R (see lines 162-180).

Line 201 – similarly to preceding comment “The point estimates are remarkably consistent;” are they consistent in some way that can be expressed with a metric?
Response: Yes. The pearson correlation is 0.96 (info added to that sentence).

Line 215 – I’d love to see a distribution of how “local” local eQTLs are. Like how far, on average, is a marker from the transcript whose variance it explains?
Response: We did not do good job of explaining this in the original methods. Each cis eQTL is based on the LOD score at the gene itself. As more clearly described on lines 740-749 of the revised ms, we first found all LOD peaks that were close to the expressed gene. Next, we shifted over to the marker closest to that gene (oftentimes this was the gene itself) and extracted the test results at that location. Thus, there really is not much (or any) distance of the test from the gene. This is why all the blue dots fall exactly on the diagonal in Fig 2.

The “Concluding remarks” include real gems of interpretation that I will return to frequently in the coming weeks. But some of this is written too colloquially. The first sentence, second clause, is missing words. And “etc.” at line 510 is too casual. Even for PLoS.
Response: Fixed.

I may have missed it, but is a link given for the scripts used in the bioinformatic and quantitative genetic analyses?

Response: We now start the Methods with a statement of how to access and use all the programs that we wrote for analysis. All standard public tools are cited in the standard way.

Minor points
Line 52 – first use of “RNASeq” should probably spell this out. Better yet, I’d lead with “Gene expression is a quantitative trait.” No need for the qualification.
Response: Revised as suggested.

Line 72 – “cis-regulatory molecules” What the heck is that? Do you mean transcription factors? Say so.
Response: Fixed.
Line 566 – how were tissues disrupted? With a bead beater or similar?

Response: Yes, detail added.

Reviewer #3: In this study, Veltsos and Kelly address the dynamics of eQTLs and their contribution to genetic variation in Mimulus guttatus via a multiparent mapping population. Ultimately, this work highlights the quantitative landscape of gene expression in leaves of a natural population of Mimulus. The authors showed that in M. guttatus a majority of the genetic variation in gene expression (two-thirds) arises from allelic diversity amongst

cis eQTLs for the Iron Mountain population. On the other hand, trans eQTLs show stronger signals of dominance compared to cis eQTLs and are correlated with multiple expressed genes, most likely functioning as network regulators. The authors have generated an impressively large and novel transcriptomic dataset that will be valuable to the community. We do, however, have some comments:

1) Given the strong cis-eQTL effects on gene expression it might be interesting to see if allelic series lead to multimodal expression patterns (see for example Hsieh et al, Genome Biology, 2007: Mixture modeling of transcript abundance classes in natural populations.

Response: Suggestion embraced. We now include two new figure panels 4C, 4D on how strong cis eQTL effects can generate multi-modal distributions. Interestingly, we often do not see obvious multi-modality because of high allelic diversity at eQTLs (the many distinct genotype specific distributions blend together). This is a nice and easily digestible addition to the results.

2) The figure legends could be greatly enhanced with increased explanations for each panel. Currently, the figures and legends alone are not sufficient to interpret all of the results without digging into the methods and results. For example, Figure 3D, what does d/a represent?

Response: We now explicitly define d/a as standardized dominance and explain how the ratio works in the legend. We have added additional explanation to all figure legends.

3) The authors could consider to present read counts on the y axis of several figure panels as relative read counts, as the number of genes expressed varies widely between species.

Response: We did not have read counts on the y-axis of Figures in the original draft. We have added text to figure legends explaining all the counts that are depicted on y-axes.

4) The study compares their findings of high allelic diversity of cis eQTLs to Liu et al., 2019. This is a relevant study that compares the contribution of cis/trans eQTLs for several species and we appreciate the broad scope. However, this comparison contains only studies on animals and yeast. If cis/trans comparisons have been made in plants, these would be beneficial for the reader. For example, work from Nathaniel Street and colleagues on selective constraint on eQTL in poplar might be relevant (Mähler et al, PLoS Genetics, 2017: Gene co-expression network connectivity is an important determinant of selective constraint) as might eQTL studies on RIL populations in Arabidopsis by Dan Kliebenstein, Maarten Koornneef, and Thomas Juenger and their colleagues (e.g., https://doi.org/10.1073/pnas.0610429104, https://doi.org/10.1105/tpc.113.115352 and https://doi.org/10.1534/genetics.106.064972).

Response: Most of the eQTL work in plants is not comparable because it uses lines from different populations or lines selected based on interesting phenotypic differences (many examples in corn and rice and Arabidopsis). However, the Mahler et al paper on poplar is a definite exception and is cited as such (lines 562-564 of revised ms). The Arabidopsis papers characterize interpopulation genetic differences, but we have included reference to this work in relation to how sequence variation in promoter regions affects expression (see lines 432-434).

5) Given this manuscript's focus on G matrix evolution there appear to be some relevant references missing, for example some of the work by Mark Blows and colleagues on G matrix evolution in Drosophila serrata (see for example Blows et al, American Naturalist, 2015: The phenome-wide distribution of genetic variance).

Response: We should have found this very relevant paper before submitting the first draft. It is now cited in the first paragraph (line 65) and also later when discussion PCA and the G matrix.

6) The order in which figure panels are introduced in the text is not always congruent with the actual order of figure panels. In addition, it appears that the supplementary tables are not listed in numerical order according to appearance in the manuscript. For example, the first supplementary table listed is #8.

Response: The numbering of supplemental tables has been revised. They now appear in order as cited in the text.

9) Lines 185 and 186: how do the combined analysis and cross-specific analysis compare to one another in terms of the numbers of cis-eQTLs identified?
Response: We added this information (see lines 201-203), but also caution why the two sets of numbers are not comparable.
10) Lines 207 and 208: VE appears high. Were any steps taken to minimize or control for environmental variation? For example, to control for circadian rhythm effects, how much time was taken for sampling within a cohort of 500 plants? Was each cohort sampled at the same or a different time of day? And were families present across multiple cohorts or kept within single cohorts? The latter might influence the combined versus the cross-specific analyses?
Response: We failed to include this essential information in the first draft. To control for control for circadian rhythm effects, we collected all leaves at mid-day, 4-6 hours after the start of the light interval (see lines 629-633 of the revised ms). Each family was grown in two cohorts and IM767 lines was grown in all four cohorts.

Limits on sequencing depth are part of the cause for our high Ve (Supplemental Figure 6 of the revised ms). For highly expressed genes, where sequencing depth is not limiting for estimation, the narrow sense heritability of expression is comparable to that of whole organism traits like flower size or days to flower or stigma anther separation (30-40%). Many papers report "broad sense heritability" of gene expression at greater than 50% but this is usually in line crosses (where the parents are divergent and every polymorphism segregates at 50/50) or among inbred lines. Even when you ignore dominance the genetic variance among inbred lines is 2 Va and nobody bothers to divide by 2 which would make the outputs more comparable to what are reporting.

11) Lines 272 and 273: how do the combined analysis and cross-specific analysis compare to one another in terms of the numbers of trans-eQTLs identified?
Response: These are exactly the same. We took all trans eQTLs from the Cross specific analysis and directly tested those locations in the Combined analysis.

13) Line 512: "at the Iron Mountain location" – this is one instance where I think that the paper could provide a little more introduction to the study system. Many potential readers will not know what "Iron Mountain" refers to?
Response: We restricted the text in "Concluding remarks" (lines 552-608) with this criticism in mind. Now we talk in general about how the population context of sampling is critical for interpretation (first paragraph) followed by a summary of the key features of the Iron Mountain population (second paragraph) to interpret the estimates that we obtain in the experiment.

8) Formatting inconsistency: Most of the text uses the format cis/trans eQTLs. However, several times in the manuscript the format cis/trans-eQTL is used (lines 69, 690, 692, 806).

12) Lines 292 and 293: I would say that the distribution for trans-eQTL is quite even, but skewed toward [4,6] for cis-eQTL?
14) Typo: Line 41: "While associations studies routinely characterize genetic…"
15) Typo: Line 60: "…genetic and environmental covariances to co-expression…"
16) Typo: Line 114, 556: denovo, requires a space, de novo, as used elsewhere in the text.
17) Typo: Line 161-162: "…can have loci identical by descent through both parents."
18) Typo: Line 163: "…formation that will makes siblings more/less…"
19) Typo: Line 180: "We chose Vg[a] to estimate the…" (missing a space)
20) Typo: Line 200: "…for cis-regulatory variation is much stronger…"
21) Typo: Line 267: "Cis eQTl…"
22) Typo: Line 272: "cis eQTLs, trans eQTLs are abundant…"
23) Typo: Line 282: " …98% of the trans eQTLs/affected gene pairs were.." (extra space)
24) Typo: Line 292: "…latter exhibit a more even distribution…"

25) Typo: Line 404: "…intermediate frequencies relative to the overall genomic…"
26) Typo: Line 414: "Multi-parental mapping populations in both plants…"
27) Typo: Line 426: "…many trans-SNPs where the minor allele…"
28) Typo: Line 466: "…own transcription by binding to their own promoter region."
29) Typo: Line 501: "…for the co-expression patterns that determine…"
30) Type: Line 529: "From one generation to the next,…" (extra space after period)

Response: For corrections 8,12,14-30, we either fixed the typo or the sentence has been rewritten to improve clarity.
